# Supplementary material for: Essential Oil Blends: The Potential of Combined Use for Respiratory Tract Infections
Source: Antibiotics (Basel). 2021 Dec 10;10(12):1517. doi: 10.3390/antibiotics10121517 (PMC8698682; doi:10.3390/antibiotics10121517)
Supplement: Supplementary file 1 [file antibiotics-10-01517-s001.zip › Publishing Content Checking-for PE-20210608-Final-EN.pdf]

Table S1. The mean MIC (n = 3) and ΣFIC values of the essential oil combinations investigated against pathogens of the respiratory tract

Essential oil combinations (common name in brackets)		Mean MIC value (mg/mL) (n = 3) and ΣFIC																	
		Staphylococcus aureus (ATCC 25924)		*Streptococcus agalactiae* (ATCC 55618)		*Streptococcus pneumoniae* (ATCC 49619)		*Streptococcus pyogenes* (ATCC 12344)		*Mycobacterium smegmatis* (ATCC 19420)		*Haemophilus influenzae* (ATCC 19418)		*Klebsiella pneumoniae* (ATCC 13883)		*Moraxella catarrhalis* (ATCC 23246)		*Cryptococcus neoformans* (ATCC 14116)	
**Essential oil 1**	**Essential oil 2**	**MIC***	**ΣFIC****	**MIC***	**ΣFIC****	**MIC***	**ΣFIC****	**MIC***	**ΣFIC****	**MIC***	**ΣFIC****	**MIC***	**ΣFIC****	**MIC***	**ΣFIC****	**MIC***	**ΣFIC****	**MIC***	**ΣFIC****
Abies balsamea (fir)	*Boswellia carterii*	2.00	*0.63*	2.00	*1.00*	4.00	4.00	3.00	1.25	2.00	*0.83*	4.00	5.33	**0.75**	*0.56*	2.00	*1.00*	**0.25**	***0.16***
	Carum carvi	2.00	*1.00*	2.00	*1.00*	2.00	1.50	8.00	3.33	2.00	*0.75*	4.00	2.67	3.00	2.25	2.00	*1.00*	**0.75**	*0.84*
	Juniperus virginiana	2.00	2.50	**1.00**	*0.75*	2.00	1.50	**0.19**	*0.53*	1.50	*0.75*	6.00	8.00	**1.00**	*0.75*	2.00	1.50	**0.25**	***0.28***
	Lavandula angustifolia	3.00	2.25	2.00	*1.00*	4.00	4.00	8.00	2.33	**1.00**	***0.50***	4.00	2.00	**1.00**	*0.83*	6.00	3.00	**0.25**	***0.16***
	Melaleuca viridiflora	2.00	*0.75*	2.00	*1.00*	4.00	4.00	**1.00**	*0.67*	2.00	*0.75*	3.00	1.19	**0.75**	*0.56*	2.00	*1.00*	**0.75**	1.59
	Myrtus communis	3.00	1.13	4.00	2.00	4.00	3.00	8.00	1.83	1.50	***0.50***	3.00	1.25	**1.00**	*0.75*	4.00	2.00	**0.25**	***0.09***
	Ocimum basilicum	**1.00**	1.25	2.00	1.50	2.00	1.50	6.00	3.00	3.00	1.13	4.00	1.67	**1.00**	*1.00*	2.00	1.50	**0.38**	*0.80*
Amyris balsamifera (amyris)	*Boswellia carterii*	**0.75**	*0.80*	**0.50**	*0.63*	6.00	27.00	**0.38**	*0.59*	**0.75**	***0.50***	**0.75**	3.75	**1.00**	***0.50***	**1.00**	*0.75*	**1.00**	1.50
	Canarium luzonicum	**0.50**	*0.63*	**0.50**	*0.63*	3.00	12.75	**0.50**	*0.83*	**0.75**	***0.47***	**0.50**	2.08	**1.00**	***0.50***	2.00	1.50	**1.00**	2.00
	Ferula galbaniflua	**1.00**	1.17	**0.50**	*0.63*	4.00	17.00	**0.38**	*0.63*	**0.75**	***0.44***	1.50	6.09	1.50	*0.75*	**1.00**	*0.75*	2.00	4.67
	Lavandula angustifolia	**0.75**	1.13	**0.50**	*0.63*	**1.00**	4.50	**0.75**	1.09	**0.50**	***0.38***	**1.00**	4.17	**1.00**	*0.58*	**1.00**	*0.75*	1.50	2.25
	Salvia officinalis	**0.75**	*1.00*	**0.25**	***0.31***	**0.50**	2.13	**0.38**	*0.69*	**0.75**	***0.47***	**1.00**	4.17	2.00	1.50	**1.00**	*0.75*	**0.25**	1.25
	Styrax benzoin	**0.25**	***0.28***	**0.25**	***0.31***	**0.75**	3.09	**0.19**	***0.31***	2.00	1.25	**0.13**	*0.53*	1.50	*0.75*	**0.50**	***0.38***	**0.50**	1.17
Boswellia carterii (frankincense)	*Cinnamomum zeylanicum*	**1.00**	*0.56*	**1.00**	1.25	2.00	1.50	**0.25**	*0.56*	1.50	*0.63*	3.00	5.00	**1.00**	*0.75*	2.00	2.50	**0.13**	***0.15***
	Citrus bergamia	2.00	***0.25***	4.00	2.00	4.00	3.00	3.00	1.25	1.50	***0.44***	6.00	7.00	2.00	*0.83*	4.00	1.25	**0.25**	***0.25***
	Citrus limon	4.00	***0.50***	2.00	*1.00*	4.00	3.00	8.00	4.00	2.00	*0.58*	2.00	2.13	4.00	1.67	2.00	1.50	**0.13**	***0.10***
	Citrus sinensis	3.00	***0.38***	4.00	2.00	4.00	3.00	8.00	6.00	2.00	*0.58*	8.00	8.50	2.00	*1.00*	2.00	*0.83*	**0.13**	***0.13***
	Commiphora molmol	2.00	***0.38***	2.00	*1.00*	4.00	4.00	**0.19**	*0.55*	1.50	*1.00*	2.00	6.00	3.00	*0.94*	2.00	*1.00*	**0.38**	*0.56*
	Elettaria cardamonum	3.00	1.69	2.00	*1.00*	4.00	3.00	2.00	*1.00*	2.00	***0.46***	4.00	4.67	1.50	1.13	2.00	*1.00*	**0.50**	***0.42***
	Ferula galbaniflua	2.00	***0.46***	**1.00**	***0.50***	3.00	2.25	**0.50**	***0.29***	8.00	2.00	3.00	3.19	2.00	*1.00*	2.00	*1.00*	**0.13**	***0.23***

	Helichrysum italicum	2.00	***0.38***	2.00	*1.00*	4.00	3.00	**0.50**	***0.29***	2.00	*0.83*	6.00	6.75	6.00	2.00	2.00	1.17	**0.19**	*0.84*
	Juniperus virginiana	1.50	1.59	**1.00**	*0.75*	2.00	1.50	**0.25**	*0.73*	3.00	1.25	3.00	6.00	1.50	*0.75*	2.00	1.50	**0.50**	*0.75*
	Lavandula angustifolia	2.00	1.13	2.00	*1.00*	4.00	4.00	8.00	3.00	1.50	*0.63*	3.00	3.50	**1.00**	*0.58*	2.00	*1.00*	**0.25**	***0.25***
	Melaleuca viridiflora	2.00	***0.38***	2.00	*1.00*	2.00	2.00	8.00	6.00	2.00	*0.58*	8.00	8.50	**1.00**	***0.50***	2.00	*1.00*	**0.50**	1.25
	Ocimum basilicum	**1.00**	1.06	2.00	1.50	4.00	3.00	2.00	1.17	2.00	*0.58*	2.00	2.17	**0.50**	***0.38***	2.00	1.50	**0.38**	*0.94*
	Origanum vulgare	2.00	***0.25***	2.00	*1.00*	4.00	3.00	3.00	2.25	**1.00**	***0.33***	2.00	2.33	**1.00**	***0.50***	2.00	*1.00*	**0.50**	***0.38***
	Pimenta racemosa	**1.00**	1.06	2.00	*1.00*	2.00	2.00	1.50	1.88	**1.00**	***0.42***	4.00	5.33	**0.50**	*0.63*	2.00	1.50	**0.25**	*0.63*
	Pinus sylvestris	2.00	*0.63*	2.00	*1.00*	2.00	2.00	8.00	4.67	2.00	***0.46***	8.00	8.50	2.00	*1.00*	2.00	*1.00*	**0.13**	*0.56*
	Piper nigrum	2.00	***0.25***	2.00	*1.00*	4.00	2.67	1.50	*0.63*	8.00	2.33	4.00	5.00	**1.00**	***0.50***	2.00	*1.00*	**0.50**	1.25
	Rosmarinus officinalis	2.00	***0.38***	2.00	*1.00*	2.00	1.50	1.50	1.13	2.00	*0.58*	8.00	8.50	2.00	*1.00*	2.00	*1.00*	**0.13**	***0.31***
	Salvia officinalis	4.00	1.58	2.00	*1.00*	2.00	1.50	8.00	6.00	**1.00**	***0.29***	8.00	9.33	**1.00**	*0.75*	4.00	2.00	**0.50**	2.25
	Santalum austrocaledonicum	**0.38**	***0.27***	**0.13**	***0.28***	**0.13**	***0.09***	**0.63**	5.16	2.00	1.67	**0.50**	2.50	3.00	*0.94*	**1.00**	2.25	**0.19**	*0.59*
	Zingiber officinale	2.00	1.13	**1.00**	*0.75*	**1.00**	*0.75*	1.50	1.13	4.00	1.67	6.00	7.00	**1.00**	***0.38***	2.00	1.50	**0.38**	*0.56*
Canarium luzonicum (elemi)	*Boswellia carterii*	3.00	*0.94*	4.00	2.00	2.00	1.50	8.00	4.67	3.00	*0.88*	2.00	2.33	8.00	4.00	2.00	*1.00*	**0.19**	***0.28***
	Citrus sinensis	3.00	*0.94*	8.00	4.00	2.00	*1.00*	8.00	6.67	8.00	2.00	2.00	***0.46***	8.00	4.00	2.00	*0.83*	**0.13**	***0.19***
	Elettaria cardamonum	2.00	1.50	8.00	4.00	2.00	*1.00*	8.00	4.67	2.00	***0.38***	2.00	*0.67*	2.00	1.50	2.00	*1.00*	**0.25**	***0.33***
	Lavandula angustifolia	1.50	1.13	8.00	4.00	2.00	1.50	8.00	3.67	2.00	*0.75*	2.00	*0.67*	1.50	*0.88*	4.00	2.00	**0.19**	***0.28***
	Rosmarinus officinalis	2.00	*0.75*	8.00	4.00	2.00	*1.00*	3.00	2.50	4.00	*1.00*	8.00	1.83	**1.00**	***0.50***	2.00	*1.00*	**0.25**	*0.75*
	Zingiber officinale	2.00	1.50	4.00	3.00	4.00	2.00	**0.50**	***0.42***	1.50	*0.56*	3.00	*1.00*	**1.00**	***0.38***	2.00	1.50	**0.38**	*0.75*
Carum carvi (caraway)	*Boswellia carterii*	2.00	*0.63*	**1.00**	***0.50***	2.00	1.50	2.00	*1.00*	**1.00**	***0.29***	8.00	10.67	2.00	*1.00*	1.50	*0.75*	**0.50**	*0.75*
	Canarium luzonicum	8.00	4.00	**1.00**	***0.50***	**1.00**	***0.50***	2.00	1.17	**1.00**	***0.25***	6.00	3.00	8.00	4.00	2.00	*1.00*	**0.75**	1.50
	Citrus sinensis	4.00	1.25	2.00	*1.00*	2.00	*1.00*	4.00	3.00	1.50	***0.38***	4.00	1.58	2.00	*1.00*	**1.00**	***0.42***	**1.00**	1.50
	Coriandrum sativum	**1.00**	1.25	**0.25**	***0.19***	**0.50**	***0.38***	**1.00**	2.25	**1.00**	***0.38***	**1.00**	1.67	**1.00**	1.25	2.00	1.17	**0.13**	2.13
	Elettaria cardamonum	1.50	1.13	**1.00**	***0.50***	**1.00**	***0.50***	6.00	3.00	**1.00**	***0.19***	8.00	4.00	3.00	2.25	**1.00**	***0.50***	**0.50**	*0.67*

	Ferula galbaniflua	4.00	1.67	**1.00**	***0.50***	2.00	*1.00*	2.00	1.17	**1.00**	***0.21***	3.00	1.19	2.00	*1.00*	2.00	*1.00*	**0.50**	1.17
	Laurus nobilis	2.00	1.50	4.00	2.00	2.00	*0.75*	2.00	*0.83*	2.00	***0.50***	8.00	3.67	8.00	4.67	1.50	***0.47***	**0.25**	***0.28***
	Lavandula angustifolia	**1.00**	*0.75*	2.00	*1.00*	2.00	1.50	4.00	1.50	**1.00**	***0.38***	4.00	2.00	3.00	1.75	2.00	*1.00*	**0.25**	***0.38***
	Ocimum basilicum	**1.00**	1.25	**1.00**	*0.75*	2.00	*1.00*	2.00	1.17	2.00	***0.50***	8.00	3.33	1.50	1.13	2.00	1.50	**0.13**	***0.38***
	Styrax benzoin	2.00	*0.75*	**1.00**	***0.50***	**1.00**	***0.38***	2.00	1.17	1.50	***0.38***	8.00	4.67	2.00	*1.00*	2.00	*1.00*	1.50	3.50
	Zingiber officinale	2.00	1.50	**0.50**	***0.38***	2.00	*1.00*	2.00	1.50	**1.00**	***0.38***	3.00	1.50	**1.00**	***0.38***	2.00	1.50	**0.50**	*1.00*
Cinnamonum camphora (camphor)	*Citrus limon*	3.00	1.69	**1.00**	***0.50***	2.00	*0.83*	4.00	2.00	**1.00**	***0.19***	6.00	3.38	4.00	2.67	2.00	1.50	**0.25**	***0.11***
	Lavandula angustifolia	2.00	2.00	**1.00**	***0.50***	2.00	1.33	2.00	*0.75*	**1.00**	***0.31***	4.00	2.67	1.50	1.25	2.00	*1.00*	**0.19**	***0.12***
	Melaleuca cajuputii	2.00	1.25	**1.00**	***0.50***	**1.00**	***0.33***	2.00	*0.75*	**1.00**	***0.19***	8.00	8.00	8.00	6.00	2.00	*1.00*	**0.38**	1.55
	Myrtus communis	2.00	1.25	**1.00**	***0.50***	2.00	*0.83*	2.00	*0.63*	1.50	***0.22***	8.00	4.67	1.50	1.13	2.00	*1.00*	**0.25**	***0.09***
	Ocimum basilicum	**1.00**	1.50	2.00	1.50	**1.00**	***0.42***	2.00	1.17	2.00	***0.38***	6.00	3.50	2.00	2.00	2.00	1.50	**0.25**	*0.53*
	Pinus sylvestris	2.00	1.50	**1.00**	***0.50***	2.00	1.33	2.00	1.17	**1.00**	***0.13***	6.00	3.38	2.00	1.50	2.00	*1.00*	**0.13**	*0.52*
	Zingiber officinale	2.00	2.00	**0.50**	***0.38***	2.00	*0.83*	**1.00**	*0.75*	**1.00**	***0.31***	2.00	1.33	**1.00**	*0.63*	2.00	1.50	**0.75**	*0.84*
Cinnamonum zeylanicum (cinnamon)	*Citrus limon*	**0.50**	***0.28***	**0.50**	*0.63*	2.00	*1.00*	**0.75**	1.69	8.00	3.00	**1.00**	*0.73*	**0.50**	***0.33***	2.00	3.00	**0.13**	***0.13***
	Citrus sinensis	2.00	1.13	**1.00**	1.25	2.00	*1.00*	**0.75**	1.88	**0.50**	***0.19***	**0.75**	*0.55*	2.00	1.50	2.00	2.33	**0.06**	***0.07***
	Commiphora molmol	**0.50**	***0.31***	2.00	2.50	2.00	1.50	**0.50**	2.33	4.00	3.00	**0.75**	2.00	**0.50**	***0.28***	2.00	2.50	**0.13**	***0.21***
	Elettaria cardamonum	**0.50**	***0.50***	**1.00**	1.25	2.00	*1.00*	**0.75**	1.69	**1.00**	***0.31***	2.00	1.67	**0.50**	***0.50***	2.00	2.50	**0.13**	***0.13***
	Eucalyptus globulus	**0.50**	***0.31***	**1.00**	1.25	2.00	1.17	**1.00**	2.25	**1.00**	***0.38***	2.00	1.67	**0.50**	***0.38***	2.00	2.50	**0.13**	***0.15***
	Ferula galbaniflua	2.00	1.33	**0.50**	*0.63*	**1.00**	***0.50***	**0.75**	1.75	**0.50**	***0.17***	**1.00**	*0.73*	2.00	1.50	2.00	2.50	**0.50**	*1.00*
	Lavandula angustifolia	**0.50**	***0.50***	**0.50**	*0.63*	2.00	1.50	**1.00**	2.13	**1.00**	***0.50***	**0.75**	*0.63*	**1.00**	*0.83*	2.00	2.50	**0.13**	***0.15***
	Melaleuca alternifolia	**1.00**	*0.63*	**1.00**	1.33	**1.00**	***0.50***	**0.31**	*0.73*	**1.00**	***0.38***	**0.75**	*0.75*	**0.50**	***0.31***	2.00	2.50	**0.06**	***0.08***
	Ocimum basilicum	**0.50**	*0.75*	**1.00**	1.50	3.00	1.50	**0.38**	*0.88*	**1.00**	***0.38***	2.00	1.50	**0.50**	***0.50***	2.00	3.00	**0.13**	***0.33***
	Pinus sylvestris	**1.00**	*0.75*	2.00	2.50	2.00	1.50	**1.00**	2.33	**1.00**	***0.31***	**1.00**	*0.73*	2.00	1.50	2.00	2.50	**0.06**	***0.29***
	Rosmarinus officinalis	**1.00**	*0.63*	2.00	2.50	2.00	*1.00*	**1.00**	2.50	**1.00**	***0.38***	**0.75**	*0.55*	2.00	1.50	2.00	2.50	**0.06**	***0.17***
	Styrax benzoin	**1.00**	*0.63*	**1.00**	1.25	2.00	*0.75*	**0.38**	*0.88*	**1.00**	***0.38***	**1.00**	*0.92*	2.00	1.50	2.00	2.50	**0.13**	***0.25***

	Thymus vulgaris	**1.00**	*0.63*	**1.00**	1.50	**0.50**	***0.38***	**1.00**	3.00	**0.50**	***0.19***	**0.38**	*0.63*	2.00	1.50	2.00	3.00	**0.06**	***0.21***
	Zingiber officinale	**0.50**	***0.50***	**0.50**	*0.75*	**1.00**	***0.50***	**0.19**	***0.47***	4.00	2.00	**0.50**	***0.42***	**0.50**	***0.31***	2.00	3.00	**0.13**	***0.21***
Citrus aurantifolia (lime)	*Rosa damascena*	2.00	*0.75*	**1.00**	***0.50***	2.00	*1.00*	1.50	6.38	**1.00**	***0.38***	2.00	1.13	8.00	4.00	1.50	*0.75*	**0.06**	***0.10***
	Rosmarinus officinalis	2.00	***0.50***	2.00	*1.00*	2.00	*1.00*	8.00	6.00	2.00	***0.50***	4.00	***0.50***	2.00	*1.00*	2.00	*1.00*	**0.13**	***0.33***
	Salvia officinalis	2.00	*0.92*	**1.00**	***0.50***	2.00	*1.00*	8.00	6.00	2.00	***0.50***	6.00	1.38	8.00	6.00	2.00	*1.00*	**0.25**	1.17
Citrus bergamia (bergamot)	*Citrus aurantifolia*	2.00	***0.38***	**1.00**	***0.50***	4.00	2.00	2.00	*0.83*	**1.00**	***0.25***	**1.00**	***0.23***	4.00	1.67	2.00	*0.63*	**0.13**	***0.15***
	Citrus limon	8.00	*1.00*	**1.00**	***0.50***	4.00	2.00	2.00	*0.83*	1.50	***0.38***	1.50	***0.34***	2.00	*0.67*	2.00	1.13	2.00	1.67
	Commiphora molmol	2.00	***0.38***	**1.00**	***0.50***	**1.00**	*0.75*	**0.38**	1.06	2.00	1.25	2.00	4.33	**0.50**	***0.11***	2.00	*0.63*	3.00	4.50
	Coriandrum sativum	**1.00**	1.06	**0.25**	***0.19***	4.00	3.00	**0.75**	1.63	**0.75**	***0.28***	**0.50**	*0.75*	**1.00**	1.17	2.00	*0.79*	**0.13**	2.06
	Cupressus sempervirens	4.00	*0.92*	2.00	*1.00*	**1.00**	***0.31***	6.00	2.00	**1.00**	***0.25***	1.50	***0.50***	2.00	*0.83*	2.00	***0.25***	**1.00**	1.50
	Eucalyptus globulus	6.00	1.13	**1.00**	***0.50***	4.00	2.33	8.00	3.33	**1.00**	***0.25***	1.50	***0.50***	2.00	*0.83*	2.00	*0.63*	2.00	2.00
	Helichrysum italicum	2.00	***0.38***	**1.00**	***0.50***	4.00	2.00	**0.75**	***0.38***	**1.00**	***0.38***	4.00	1.17	4.00	*1.00*	2.00	*0.79*	**0.19**	*0.84*
	Lavandula angustifolia	2.00	1.13	**1.00**	***0.50***	4.00	3.00	2.00	*0.58*	**1.00**	***0.38***	1.50	***0.50***	1.50	*0.75*	2.00	*0.63*	**0.75**	*0.75*
	Lavandula burnati	2.00	*0.79*	**1.00**	***0.50***	4.00	3.00	2.00	1.33	2.00	*0.75*	4.00	1.67	**1.00**	*0.67*	2.00	*0.63*	**0.75**	*0.63*
	Lavandula spica	2.00	1.13	**1.00**	***0.50***	4.00	2.33	2.00	*0.67*	**1.00**	***0.25***	1.50	***0.44***	1.50	*0.75*	2.00	1.13	**0.25**	***0.21***
	Melaleuca alternifolia	2.00	***0.38***	**1.00**	*0.58*	**1.00**	***0.50***	4.00	2.00	**1.00**	***0.25***	1.50	*0.75*	2.00	*0.58*	4.00	1.25	**0.50**	*0.58*
	Myrtus communis	2.00	***0.38***	2.00	*1.00*	**1.00**	***0.50***	8.00	1.83	**1.00**	***0.21***	1.50	***0.38***	1.50	*0.63*	2.00	*0.63*	**0.25**	***0.19***
	Origanum vulgare	8.00	*1.00*	**1.00**	***0.50***	**1.00**	***0.50***	8.00	5.33	**1.00**	***0.29***	6.00	2.00	8.00	3.33	2.00	*0.63*	**0.25**	***0.19***
	Pinus sylvestris	1.50	***0.47***	2.00	*1.00*	**1.00**	*0.75*	8.00	4.00	**1.00**	***0.19***	3.00	*0.69*	**1.00**	***0.42***	4.00	1.25	2.00	9.00
	Rosa damascena	**1.00**	***0.31***	**1.00**	***0.50***	**1.00**	***0.50***	1.50	6.25	8.00	3.00	1.50	*1.00*	3.00	1.25	**1.00**	***0.31***	**0.13**	***0.19***
	Rosmarinus officinalis	2.00	***0.38***	**1.00**	***0.50***	2.00	*1.00*	8.00	5.33	2.00	***0.50***	**0.75**	***0.17***	**0.75**	***0.31***	**1.00**	***0.31***	1.50	3.75
	Salvia officinalis	2.00	*0.79*	**1.00**	***0.50***	8.00	4.00	8.00	5.33	**1.00**	***0.25***	3.00	*1.00*	**1.00**	*0.67*	**1.00**	***0.31***	**0.19**	*0.84*
	Santalum austrocaledonicum	1.50	1.09	**0.50**	1.13	**1.00**	***0.50***	**0.63**	5.10	**0.19**	***0.15***	**0.19**	*0.78*	**1.00**	***0.23***	4.00	8.25	**0.25**	*0.79*
	Thymus vulgaris	2.00	***0.38***	**1.00**	*0.75*	**1.00**	*0.75*	**1.00**	1.17	**1.00**	***0.25***	1.50	1.75	2.00	*0.83*	2.00	1.13	**0.13**	***0.40***

Citrus limon (lemon)	*Citrus sinensis*	2.00	***0.25***	2.00	*1.00*	4.00	2.00	3.00	2.25	1.50	***0.38***	6.00	*0.75*	2.00	*0.83*	4.00	2.67	**1.00**	*0.83*
	Commiphora molmol	2.00	***0.38***	**1.00**	***0.50***	4.00	3.00	2.00	5.83	4.00	2.50	4.00	8.25	**1.00**	***0.23***	2.00	1.50	**0.50**	*0.67*
	Melaleuca viridiflora	2.00	***0.38***	**1.00**	***0.50***	2.00	1.50	**1.00**	*0.75*	2.00	***0.50***	4.00	***0.50***	3.00	1.25	2.00	1.50	**1.00**	2.33
	Mentha piperita	2.00	1.13	2.00	*1.00*	2.00	*0.75*	1.50	*0.88*	**1.00**	***0.25***	6.00	1.88	4.00	2.67	2.00	1.50	**0.25**	***0.17***
	Pimenta racemosa	2.00	2.13	**1.00**	***0.50***	2.00	1.50	**0.75**	*0.94*	2.00	*0.75*	4.00	1.58	**0.75**	*0.88*	2.00	2.00	**0.25**	*0.58*
	Rosa damascena	2.00	*0.63*	**1.00**	***0.50***	2.00	*1.00*	**0.50**	2.13	**1.00**	***0.38***	3.00	1.69	2.00	*0.83*	2.00	1.50	**0.13**	***0.17***
	Santalum austrocaledonicum	**0.38**	***0.27***	**0.13**	***0.28***	**0.25**	***0.13***	**0.19**	1.55	2.00	1.58	**0.50**	2.03	4.00	*0.92*	**1.00**	2.50	**0.06**	***0.19***
	Thymus vulgaris	2.00	***0.38***	**1.00**	*0.75*	**1.00**	*0.75*	2.00	2.50	1.50	***0.38***	4.00	4.25	8.00	3.33	2.00	2.00	**0.50**	1.50
Citrus sinensis (orange)	*Melaleuca alternifolia*	3.00	*0.56*	**1.00**	*0.58*	**1.00**	***0.50***	3.00	2.50	2.00	***0.50***	4.00	1.58	4.00	1.50	2.00	*0.83*	**0.50**	*0.58*
	Pimenta racemosa	1.50	1.59	**1.00**	***0.50***	2.00	1.50	1.50	2.25	2.00	*0.75*	4.00	1.58	**0.50**	*0.63*	2.00	1.33	**0.50**	1.25
	Rosa damascena	1.50	***0.47***	**1.00**	***0.50***	2.00	*1.00*	**0.75**	3.38	**1.00**	***0.38***	6.00	3.38	4.00	2.00	2.00	*0.83*	**0.13**	***0.19***
	Rosmarinus officinalis	2.00	***0.38***	2.00	*1.00*	4.00	2.00	2.00	2.00	2.00	***0.50***	8.00	*1.00*	2.00	*1.00*	2.00	*0.83*	**0.13**	***0.31***
	Salvia officinalis	8.00	3.17	2.00	*1.00*	4.00	2.00	1.50	1.50	2.00	***0.50***	8.00	1.83	**0.50**	***0.38***	2.00	*0.83*	**1.00**	4.50
	Thymus vulgaris	**1.00**	***0.19***	**1.00**	*0.75*	**1.00**	*0.75*	2.00	3.00	2.00	***0.50***	4.00	4.25	**1.00**	***0.50***	2.00	1.33	**0.50**	1.58
Commiphora molmol (myrrh)	*Mentha piperita*	2.00	1.25	2.00	*1.00*	2.00	1.25	1.50	4.50	**1.00**	*0.63*	8.00	18.00	1.50	*0.84*	**1.00**	***0.50***	**0.50**	*0.67*
	Pinus sylvestris	2.00	*0.75*	2.00	*1.00*	2.00	2.00	1.50	4.50	1.50	*0.84*	8.00	16.50	**1.00**	***0.31***	**1.00**	***0.50***	**0.38**	1.88
	Santalum austrocaledonicum	**0.25**	***0.20***	**0.25**	*0.56*	**0.25**	***0.19***	2.00	21.33	**0.75**	*0.88*	2.00	12.00	2.00	***0.25***	2.00	4.50	**0.09**	***0.34***
	Thymus vulgaris	**1.00**	***0.25***	**1.00**	*0.75*	4.00	4.00	**1.00**	3.67	**0.50**	***0.31***	2.00	6.00	**1.00**	***0.31***	**1.00**	*0.75*	**0.25**	*0.92*
Coriandrum sativum (coriander)	*Boswellia carterii*	3.00	3.19	2.00	1.50	**1.00**	*1.00*	3.00	6.75	**1.00**	***0.42***	**0.50**	1.17	**1.00**	1.25	2.00	1.17	**1.00**	16.50
	Cinnamonum zeylanicum	**0.50**	*0.75*	**0.13**	***0.19***	**1.00**	*0.75*	**0.38**	1.50	**0.50**	***0.25***	**0.50**	*1.00*	**1.00**	1.50	2.00	2.67	**0.13**	2.08
	Citrus limon	**1.00**	1.06	4.00	3.00	4.00	3.00	**1.00**	2.25	**1.00**	***0.38***	2.00	2.79	2.00	2.33	2.00	1.67	**0.13**	2.04
	Citrus sinensis	**1.00**	1.06	2.00	1.50	4.00	3.00	1.50	3.75	**1.00**	***0.38***	4.00	5.58	2.00	2.50	2.00	*1.00*	**0.13**	2.06
	Cupressus sempervirens	4.00	4.67	2.00	1.50	**1.00**	*0.56*	**0.50**	1.08	**1.00**	***0.38***	**1.00**	1.50	**0.75**	*0.94*	2.00	*0.79*	**0.25**	4.25
	Cymbopogon citratus	**0.50**	*0.75*	**1.00**	*0.75*	**0.75**	*0.75*	**0.19**	*0.63*	**1.00**	***0.50***	2.00	3.33	**1.00**	1.06	2.00	1.17	**0.13**	2.01

	Ferula galbaniflua	2.00	2.33	2.00	1.50	**1.00**	*0.75*	**0.13**	***0.29***	**1.00**	***0.33***	**0.50**	*0.70*	1.50	1.88	2.00	1.17	**0.19**	3.25
	Melaleuca viridiflora	1.50	1.69	**1.00**	*0.75*	2.00	2.00	**0.75**	1.88	2.00	*0.75*	3.00	4.19	**1.00**	1.25	2.00	1.17	**0.09**	1.69
	Myrtus communis	**1.00**	1.13	**1.00**	*0.75*	2.00	1.50	**0.75**	1.55	1.50	***0.50***	2.00	2.83	**1.00**	1.25	2.00	1.17	**0.09**	1.53
	Pinus sylvestris	**1.00**	1.25	2.00	1.50	4.00	4.00	**1.00**	2.33	**1.00**	***0.31***	4.00	5.58	2.00	2.50	2.00	1.17	**0.06**	1.26
	Piper nigrum	**1.00**	1.06	4.00	3.00	1.50	*1.00*	**1.00**	2.17	**1.00**	***0.38***	**1.00**	1.58	2.00	2.50	2.00	1.17	**0.09**	1.69
	Salvia officinalis	**1.00**	1.33	4.00	3.00	2.00	1.50	**0.38**	*0.94*	**0.50**	***0.19***	6.00	9.00	1.50	2.25	2.00	1.17	**0.06**	1.26
	Santalum austrocaledonicum	**0.75**	1.25	**0.25**	*0.63*	**0.38**	***0.28***	**0.63**	6.25	**0.38**	***0.34***	**0.50**	2.67	**1.00**	1.06	2.00	4.67	**0.13**	2.33
	Zingiber officinale	**0.50**	*0.75*	2.00	2.00	2.00	1.50	**0.19**	***0.47***	**0.75**	***0.38***	2.00	3.00	**1.00**	1.13	2.00	1.67	**0.13**	2.13
Cupressus sempervirens (cypress)	*Boswellia carterii*	2.00	***0.46***	2.00	*1.00*	1.50	*0.84*	8.00	3.33	2.00	*0.58*	2.00	2.33	**1.00**	***0.50***	2.00	*0.63*	**0.25**	***0.38***
	Citrus limon	4.00	*0.92*	2.00	*1.00*	1.50	***0.47***	8.00	3.33	2.00	***0.50***	6.00	1.38	8.00	3.33	2.00	1.13	**0.38**	***0.50***
	Citrus sinensis	4.00	*0.92*	2.00	*1.00*	2.00	*0.63*	**0.63**	***0.42***	3.00	*0.75*	3.00	*0.69*	8.00	4.00	2.00	***0.46***	**0.25**	***0.38***
	Commiphora molmol	2.00	*0.58*	2.00	*1.00*	2.00	1.13	6.00	17.00	**1.00**	*0.63*	4.00	8.67	1.50	***0.47***	2.00	*0.63*	**0.50**	*1.00*
	Hyssopus officinalis	2.00	*0.83*	2.00	*1.00*	**1.00**	***0.31***	1.50	*1.00*	1.50	***0.38***	2.00	***0.46***	**1.00**	***0.50***	2.00	*0.63*	**0.50**	2.50
	Juniperus virginiana	**1.00**	1.17	2.00	1.50	**0.50**	***0.16***	**0.13**	***0.35***	2.00	*0.75*	3.00	3.50	8.00	4.00	2.00	1.13	**0.50**	*1.00*
	Lavandula angustifolia	2.00	1.33	2.00	*1.00*	1.50	*0.84*	8.00	2.33	2.00	*0.75*	4.00	1.33	**1.00**	*0.58*	2.00	*0.63*	**0.13**	***0.19***
	Melaleuca alternifolia	4.00	1.17	**1.00**	*0.58*	2.00	*0.63*	1.50	*0.75*	**1.00**	***0.25***	3.00	1.50	4.00	1.50	2.00	*0.63*	**0.09**	***0.16***
	Mentha piperita	2.00	1.33	2.00	*1.00*	2.00	***0.38***	4.00	2.00	3.00	*0.75*	2.00	*0.83*	1.50	1.13	2.00	*0.63*	**0.19**	***0.25***
	Origanum vulgare	2.00	***0.46***	2.00	*1.00*	2.00	*0.63*	6.00	4.00	2.00	*0.58*	8.00	2.67	1.50	*0.75*	2.00	*0.63*	**0.75**	*0.94*
	Pinus sylvestris	2.00	*0.83*	2.00	*1.00*	2.00	1.13	8.00	4.00	4.00	*0.75*	2.00	***0.46***	8.00	4.00	2.00	*0.63*	**0.13**	*0.63*
	Rosmarinus officinalis	2.00	*0.58*	**1.00**	***0.50***	2.00	*0.63*	8.00	5.33	2.00	***0.50***	8.00	1.83	8.00	4.00	2.00	*0.63*	**0.13**	***0.38***
	Salvia officinalis	2.00	*1.00*	**1.00**	***0.50***	8.00	2.50	8.00	5.33	2.00	***0.50***	8.00	2.67	**1.00**	*0.75*	2.00	*0.63*	**0.25**	1.25
	Salvia sclarea	2.00	***0.46***	2.00	*1.00*	2.00	***0.25***	4.00	1.33	2.00	***0.50***	2.00	*0.67*	**1.00**	***0.31***	2.00	***0.25***	**0.25**	1.25
	Santalum austrocaledonicum	**0.19**	***0.16***	**0.13**	***0.28***	**0.09**	***0.03***	**0.19**	1.53	2.00	1.58	4.00	16.67	8.00	2.50	2.00	4.13	**0.13**	***0.46***
	Citrus sinensis	**1.00**	*0.56*	**1.00**	***0.50***	**1.00**	*0.75*	**1.00**	1.83	2.00	*0.75*	2.00	*0.79*	**1.00**	***0.31***	2.00	*0.83*	**0.25**	***0.14***

Cymbopogon citratus (lemongrass)	*Melaleuca alternifolia*	**1.00**	*0.63*	**0.50**	***0.29***	**1.00**	*0.75*	**1.00**	1.67	**1.00**	***0.38***	3.00	2.00	2.00	***0.38***	2.00	*1.00*	**0.25**	***0.18***
	Melaleuca viridiflora	**1.00**	*0.63*	**0.50**	***0.25***	**1.00**	*1.00*	**0.75**	1.38	1.50	*0.56*	2.00	*0.79*	2.00	*0.63*	2.00	*1.00*	**0.25**	*0.52*
	Mentha piperita	**1.00**	*1.00*	**1.00**	***0.50***	**1.00**	*0.63*	**1.00**	1.67	**1.00**	***0.38***	2.00	1.17	2.00	1.13	4.00	2.00	**0.25**	***0.10***
	Pimenta racemosa	**1.00**	1.50	**1.00**	***0.50***	**1.00**	*1.00*	**1.00**	2.33	**1.00**	***0.50***	2.00	1.33	**1.00**	1.06	2.00	1.50	**0.13**	***0.26***
	Rosmarinus officinalis	**1.00**	*0.63*	**0.75**	***0.38***	**1.00**	*0.75*	**1.00**	1.83	**1.00**	***0.38***	4.00	1.58	2.00	*0.63*	2.00	*1.00*	**0.13**	***0.26***
	Santalum austrocaledonicum	**0.50**	*0.58*	**0.13**	***0.28***	**0.13**	***0.09***	**0.25**	2.33	**1.00**	*0.92*	**0.50**	2.17	**1.00**	***0.13***	2.00	4.50	**0.13**	***0.34***
	Thymus vulgaris	**1.00**	*0.63*	**0.50**	***0.38***	**0.50**	***0.50***	**1.00**	2.33	3.00	1.13	3.00	4.00	**0.50**	***0.16***	**1.00**	*0.75*	**0.13**	***0.34***
Daucus carota (carrot seed)	*Citrus aurantifolia*	4.00	1.50	**1.00**	***0.50***	2.00	1.50	2.00	1.50	**1.00**	***0.25***	1.50	3.09	2.00	*1.00*	2.00	*1.00*	**0.38**	*0.75*
	Citrus bergamia	8.00	2.50	**0.50**	***0.25***	2.00	1.50	2.00	1.33	**1.00**	***0.25***	2.00	4.33	3.00	1.25	2.00	*0.63*	1.50	2.75
	Citrus limon	8.00	2.50	2.00	*1.00*	2.00	1.50	2.00	1.50	**1.00**	***0.25***	**1.00**	2.06	2.00	*0.83*	1.50	1.13	**0.75**	1.25
	Citrus sinensis	4.00	1.25	**1.00**	***0.50***	2.00	1.50	2.00	2.00	2.00	***0.50***	2.00	4.13	2.00	*1.00*	2.00	*0.83*	**0.75**	1.38
	Lavandula angustifolia	**1.00**	*0.75*	**1.00**	***0.50***	2.00	2.00	2.00	1.25	**1.00**	***0.38***	1.50	3.25	3.00	1.75	2.00	*1.00*	**0.19**	***0.34***
	Rosmarinus officinalis	2.00	*0.75*	**1.00**	***0.50***	2.00	1.50	2.00	2.00	**1.00**	***0.25***	2.00	4.13	2.00	*1.00*	1.50	*0.75*	**0.50**	1.67
Elettaria cardamonum (cardamom)	*Citrus limon*	2.00	1.13	2.00	*1.00*	2.00	*1.00*	2.00	*1.00*	**1.00**	***0.19***	8.00	1.83	**1.00**	*0.67*	2.00	1.50	**0.75**	***0.50***
	Coriandrum sativum	**0.50**	*0.75*	**0.25**	***0.19***	**0.50**	***0.38***	**0.75**	1.69	**1.00**	***0.31***	**0.50**	*0.75*	**1.00**	1.50	2.00	1.17	**0.13**	2.04
	Myrtus communis	1.50	*0.94*	2.00	*1.00*	2.00	*1.00*	4.00	1.25	1.50	***0.22***	8.00	2.00	**1.00**	*0.75*	2.00	*1.00*	**0.25**	***0.15***
	Pinus sylvestris	**1.00**	*0.75*	2.00	*1.00*	2.00	1.50	8.00	4.67	1.50	***0.19***	3.00	*0.69*	3.00	2.25	2.00	*1.00*	**0.19**	*0.81*
Eucalyptus globulus (eucalyptus)	*Citrus limon*	2.00	***0.38***	2.00	*1.00*	2.00	1.17	8.00	4.00	2.00	***0.50***	8.00	1.83	8.00	3.33	2.00	1.50	**0.13**	***0.10***
	Commiphora molmol	2.00	***0.50***	2.00	*1.00*	**0.50**	***0.42***	**0.63**	1.82	**1.00**	*0.63*	8.00	17.33	2.00	*0.63*	2.00	*1.00*	**0.25**	***0.38***
	Coriandrum sativum	1.50	1.69	**0.50**	***0.38***	**1.00**	*0.83*	1.50	3.38	2.00	*0.75*	2.00	3.00	3.00	3.75	**1.00**	*0.58*	**0.13**	2.06
	Cupressus sempervirens	2.00	*0.58*	1.50	*0.75*	1.50	*0.59*	8.00	3.33	2.00	***0.50***	8.00	2.67	**1.00**	***0.50***	2.00	*0.63*	**0.25**	***0.38***
	Cymbopogon citratus	**1.00**	*0.63*	**1.00**	***0.50***	2.00	1.67	**0.13**	***0.20***	**1.00**	***0.38***	3.00	1.50	8.00	2.50	2.00	*1.00*	**0.06**	***0.04***
	Juniperus virginiana	**1.00**	1.13	**1.00**	*0.75*	2.00	1.17	**0.25**	*0.73*	**1.00**	***0.38***	3.00	3.50	**1.00**	***0.50***	2.00	1.50	**0.13**	***0.19***
	Lavandula angustifolia	**1.00**	*0.63*	2.00	*1.00*	2.00	*1.00*	8.00	3.00	2.00	*0.75*	8.00	2.67	**1.00**	*0.58*	2.00	*1.00*	**0.19**	***0.19***

	Melaleuca alternifolia	2.00	***0.50***	**1.00**	*0.58*	2.00	1.17	3.00	1.75	1.50	***0.38***	2.00	*1.00*	4.00	1.50	2.00	*1.00*	**0.06**	***0.07***
	Mentha piperita	2.00	1.25	2.00	*1.00*	1.50	*0.69*	8.00	4.67	2.00	***0.50***	8.00	3.33	**1.00**	*0.75*	2.00	*1.00*	**0.13**	***0.10***
	Origanum vulgare	3.00	*0.56*	2.00	*1.00*	4.00	2.33	8.00	6.00	2.00	*0.58*	8.00	2.67	**1.00**	***0.50***	2.00	*1.00*	**0.19**	***0.14***
	Pinus sylvestris	2.00	*0.75*	2.00	*1.00*	**1.00**	*0.83*	8.00	4.67	2.00	***0.38***	8.00	1.83	2.00	*1.00*	2.00	*1.00*	**0.06**	***0.28***
	Rosmarinus officinalis	2.00	***0.50***	2.00	*1.00*	2.00	1.17	8.00	6.00	2.00	***0.50***	8.00	1.83	1.50	*0.75*	2.00	*1.00*	**0.25**	*0.63*
	Styrax benzoin	8.00	2.00	8.00	4.00	2.00	*0.92*	8.00	4.67	4.00	*1.00*	2.00	*0.83*	8.00	4.00	2.00	*1.00*	8.00	14.67
	Thymus vulgaris	2.00	***0.50***	2.00	1.50	2.00	1.67	2.00	2.50	**1.00**	***0.25***	6.00	7.00	2.00	*1.00*	2.00	1.50	**0.13**	***0.40***
	Zingiber officinale	2.00	1.25	2.00	1.50	2.00	1.17	**0.25**	***0.19***	**1.00**	***0.38***	4.00	1.33	2.00	*0.75*	2.00	1.50	**0.50**	*0.75*
Ferula galbaniflua (galbanum)	*Canarium luzonicum*	2.00	*0.83*	2.00	*1.00*	2.00	*1.00*	**1.00**	*0.67*	2.00	***0.42***	4.00	*0.92*	**1.00**	***0.50***	4.00	2.00	**0.25**	*0.58*
	Commiphora molmol	**1.00**	***0.29***	2.00	*1.00*	2.00	1.50	**0.50**	1.50	1.50	*0.88*	2.00	4.13	2.00	*0.63*	4.00	2.00	**0.25**	*0.58*
	Elettaria cardamonum	2.00	1.33	8.00	4.00	2.00	*1.00*	6.00	3.50	3.00	***0.44***	6.00	1.38	**0.75**	*0.56*	8.00	4.00	**0.75**	1.25
	Melaleuca viridiflora	2.00	*0.58*	2.00	*1.00*	2.00	1.50	**1.00**	*0.83*	2.00	***0.42***	4.00	***0.50***	2.00	*1.00*	3.00	1.50	**0.75**	2.50
	Pimenta racemosa	1.50	1.75	2.00	*1.00*	2.00	1.50	**0.38**	***0.50***	1.50	***0.50***	3.00	1.19	**0.50**	*0.63*	2.00	1.50	**0.38**	1.25
	Pinus sylvestris	4.00	1.67	2.00	*1.00*	2.00	1.50	**0.75**	***0.50***	1.50	***0.22***	4.00	***0.50***	6.00	3.00	8.00	4.00	**0.38**	2.00
	Rosa damascena	2.00	*0.83*	2.00	*1.00*	2.00	*1.00*	**1.00**	4.33	**1.00**	***0.33***	3.00	1.69	2.00	*1.00*	4.00	2.00	**0.25**	*0.58*
	Tagetes minuta	1.50	*1.00*	2.00	*1.00*	2.00	*0.75*	**0.75**	*1.00*	1.50	***0.50***	8.00	1.83	1.50	1.13	4.00	2.00	**0.25**	***0.46***
	Zingiber officinale	2.00	1.33	2.00	1.50	2.00	*1.00*	**0.25**	***0.21***	2.00	*0.67*	8.00	1.83	**1.00**	***0.38***	2.00	1.50	**0.25**	*0.58*
Foeniculum vulgare (fennel)	*Citrus limon*	2.00	1.13	8.00	4.00	8.00	6.00	8.00	3.33	3.00	*0.88*	8.00	1.83	**1.00**	*0.67*	2.00	1.50	**0.38**	***0.22***
	Hyssopus officinalis	2.00	1.50	2.00	*1.00*	3.00	2.25	3.00	2.00	2.00	*0.58*	3.00	*0.69*	1.50	1.13	2.00	*1.00*	**0.25**	1.06
	Lavandula angustifolia	2.00	2.00	8.00	4.00	8.00	8.00	8.00	2.33	3.00	1.25	8.00	2.67	2.00	1.67	2.00	*1.00*	**0.13**	***0.09***
	Melaleuca viridiflora	3.00	1.88	2.00	*1.00*	2.00	2.00	6.00	4.00	2.00	*0.58*	4.00	*0.92*	**1.00**	*0.75*	1.50	*0.75*	**0.19**	***0.42***
	Ocimum basilicum	2.00	3.00	8.00	6.00	8.00	6.00	2.00	*1.00*	3.00	*0.88*	8.00	2.00	**1.00**	*1.00*	2.00	1.50	**0.13**	***0.28***
	Origanum vulgare	2.00	2.00	2.00	*1.00*	2.00	1.25	4.00	2.67	2.00	*0.58*	6.00	2.00	**1.00**	*0.75*	2.00	*1.00*	**0.19**	***0.14***
	Rosa damascena	2.00	1.50	2.00	*1.00*	4.00	3.00	**0.75**	3.13	4.00	1.67	4.00	2.67	2.00	1.50	2.00	*1.00*	8.00	10.00
	Rosmarinus officinalis	8.00	5.00	8.00	4.00	8.00	6.00	8.00	5.33	8.00	2.33	8.00	1.83	**1.00**	*0.75*	2.00	*1.00*	**0.25**	*0.56*

	Santalum austrocaledonicum	**0.50**	*0.58*	**0.25**	*0.56*	**0.50**	***0.38***	**0.13**	1.02	**0.50**	***0.42***	1.50	6.25	**1.00**	*0.56*	2.00	4.50	**0.19**	*0.55*
Helichrysum italicum (immortelle)	*Citrus sinensis*	4.00	*0.75*	**1.00**	***0.50***	2.00	*1.00*	**0.38**	***0.31***	2.00	*0.75*	8.00	1.50	4.00	1.33	2.00	*1.00*	**0.13**	*0.56*
	Lavandula angustifolia	2.00	1.25	**1.00**	***0.50***	2.00	1.50	**0.75**	***0.34***	2.00	*1.00*	**1.00**	***0.29***	**1.00**	***0.42***	3.00	1.75	**0.38**	1.69
	Lavandula burnati	2.00	*0.92*	**1.00**	***0.50***	2.00	1.50	**0.75**	*0.63*	2.00	*1.00*	4.00	1.50	**0.50**	***0.29***	2.00	1.17	**0.38**	1.63
	Lavandula spica	**1.00**	*0.63*	**1.00**	***0.50***	2.00	1.17	**0.50**	***0.25***	2.00	*0.75*	6.00	1.50	**0.75**	***0.31***	2.00	1.67	**0.50**	2.17
	Rosa damascena	**1.00**	***0.38***	**1.00**	***0.50***	2.00	*1.00*	**0.50**	2.17	2.00	*1.00*	4.00	2.50	2.00	*0.67*	4.00	2.33	**0.06**	***0.31***
Hyssopus officinalis (hyssop)	*Citrus limon*	2.00	*0.63*	2.00	*1.00*	4.00	2.00	2.00	1.50	2.00	***0.50***	4.00	***0.50***	**1.00**	***0.42***	4.00	3.00	**0.25**	1.08
	Citrus sinensis	4.00	1.25	2.00	*1.00*	4.00	2.00	**1.00**	*1.00*	2.00	***0.50***	4.00	***0.50***	4.00	2.00	4.00	1.67	**0.13**	*0.56*
	Lavandula angustifolia	2.00	1.50	2.00	*1.00*	4.00	3.00	6.00	3.75	2.00	*0.75*	4.00	*0.92*	**0.50**	***0.29***	4.00	2.00	**0.25**	1.13
	Rosmarinus officinalis	2.00	*0.75*	2.00	*1.00*	3.00	1.50	**1.00**	*1.00*	1.50	***0.38***	4.00	***0.50***	4.00	2.00	2.00	*1.00*	**0.09**	*0.56*
	Salvia officinalis	2.00	1.17	2.00	*1.00*	3.00	1.50	**1.00**	*1.00*	2.00	***0.50***	8.00	1.83	**0.50**	***0.38***	2.00	*1.00*	**0.25**	2.00
	Santalum austrocaledonicum	**0.50**	***0.46***	**0.25**	*0.56*	**0.19**	***0.09***	**0.63**	5.31	1.50	1.19	2.00	8.13	**1.00**	***0.31***	**1.00**	2.25	**0.25**	1.67
Illicium verum (star anise)	*Carum carvi*	2.00	1.50	2.00	*1.00*	2.00	*1.00*	2.00	1.50	2.00	1.58	4.00	2.67	**0.50**	***0.38***	2.00	*1.00*	**0.50**	*0.56*
	Coriandrum sativum	**1.00**	1.50	**0.25**	***0.19***	**0.50**	***0.38***	**0.50**	1.25	2.00	1.83	2.00	3.33	**0.75**	1.13	2.00	1.17	**0.13**	2.02
	Cupressus sempervirens	2.00	1.33	2.00	*1.00*	2.00	*0.63*	2.00	1.33	2.00	1.58	3.00	1.50	**1.00**	*0.75*	2.00	*0.63*	2.00	2.25
	Elettaria cardamonum	2.00	2.00	2.00	*1.00*	2.00	*1.00*	3.00	2.25	2.00	1.46	4.00	2.00	**0.50**	***0.50***	**0.75**	***0.38***	**0.50**	***0.23***
	Foeniculum vulgare	2.00	2.00	2.00	*1.00*	2.00	1.50	2.00	1.33	2.00	1.67	4.00	2.00	**1.00**	*1.00*	2.00	*1.00*	**0.50**	***0.19***
Juniperus virginiana (cedarwood)	*Cinnamonum zeylanicum*	**1.00**	1.50	**0.50**	*0.75*	**1.00**	***0.50***	**0.25**	1.17	**0.50**	***0.25***	**0.50**	*0.83*	1.50	1.13	2.00	3.00	**0.06**	***0.10***
	Citrus bergamia	2.00	2.13	**1.00**	*0.75*	2.00	*1.00*	**0.50**	1.42	**0.50**	***0.19***	2.00	2.33	4.00	1.67	**1.00**	*0.56*	**0.50**	*0.75*
	Citrus limon	**1.00**	1.06	**1.00**	*0.75*	2.00	*1.00*	2.00	5.83	2.00	*0.75*	**1.00**	1.06	2.00	*0.83*	4.00	4.00	**0.13**	***0.17***
	Commiphora molmol	**1.00**	1.13	**1.00**	*0.75*	2.00	1.50	**0.75**	4.00	**1.00**	*0.75*	**0.75**	2.25	3.00	*0.94*	2.00	1.50	8.00	16.00
	Cymbopogon citratus	**0.50**	*0.75*	**0.50**	***0.38***	2.00	1.50	**0.75**	3.00	**0.50**	***0.25***	**0.50**	*0.67*	2.00	*0.63*	4.00	3.00	**0.09**	***0.10***
	Lavandula angustifolia	**1.00**	1.50	**0.75**	*0.56*	2.00	1.50	8.00	22.33	**1.00**	***0.50***	1.50	1.75	4.00	2.33	2.00	1.50	**0.19**	***0.28***
	Mentha piperita	**1.00**	1.50	**0.50**	***0.38***	2.00	*0.75*	**0.38**	1.13	**0.75**	***0.28***	**0.50**	*0.63*	2.00	1.50	2.00	1.50	**0.25**	***0.33***

	Origanum vulgare	**1.00**	1.06	**1.00**	*0.75*	2.00	*1.00*	1.50	4.75	**1.00**	***0.42***	2.00	2.33	8.00	4.00	2.00	1.50	**0.13**	***0.16***
	Pinus sylvestris	**1.00**	1.25	**1.00**	*0.75*	2.00	1.50	**1.00**	3.00	**1.00**	***0.31***	**1.00**	1.06	4.00	2.00	2.00	1.50	**0.06**	***0.31***
	Rosa damascena	**0.50**	*0.63*	**0.50**	***0.38***	2.00	*1.00*	1.50	10.00	**0.50**	***0.25***	**0.75**	1.13	2.00	*1.00*	2.00	1.50	**0.06**	***0.13***
	Rosmarinus officinalis	**1.00**	1.13	**1.00**	*0.75*	4.00	2.00	**0.75**	2.38	**1.00**	***0.38***	**0.50**	*0.53*	2.00	*1.00*	2.00	1.50	**0.09**	***0.28***
	Santalum austrocaledonicum	**0.25**	***0.42***	**0.50**	1.25	2.00	*1.00*	**0.25**	2.67	**0.25**	***0.23***	**0.06**	***0.32***	3.00	*0.94*	2.00	5.00	**0.50**	1.83
	Styrax benzoin	**1.00**	1.13	**0.50**	***0.38***	**1.00**	***0.38***	**0.38**	1.13	**0.50**	***0.19***	1.50	1.88	4.00	2.00	2.00	1.50	**0.25**	*0.58*
	Thymus vulgaris	**1.00**	1.13	**0.50**	***0.50***	**1.00**	*0.75*	**0.50**	1.83	**0.75**	***0.28***	**0.25**	***0.50***	3.00	1.50	2.00	2.00	**0.13**	***0.46***
	Zingiber officinale	1.50	2.25	**0.75**	*0.75*	2.00	*1.00*	2.00	6.33	**0.75**	***0.38***	**1.00**	1.17	**1.00**	***0.38***	4.00	4.00	**0.50**	*1.00*
Laurus nobilis (bay)	*Citrus limon*	2.00	1.13	8.00	4.00	8.00	3.00	8.00	3.33	2.00	***0.50***	3.00	*0.56*	3.00	1.50	2.00	1.13	**0.13**	***0.06***
	Citrus sinensis	2.00	1.13	8.00	4.00	8.00	3.00	8.00	5.33	2.00	***0.50***	6.00	1.13	2.00	1.17	2.00	***0.46***	**0.13**	***0.08***
	Coriandrum sativum	**0.50**	*0.75*	**0.13**	***0.09***	**1.00**	*0.63*	**0.75**	1.63	**1.00**	***0.38***	**0.19**	***0.27***	**1.00**	1.33	4.00	1.58	**0.06**	1.01
	Eucalyptus globulus	**1.00**	*0.63*	4.00	2.00	8.00	3.67	**0.63**	***0.26***	2.00	***0.50***	**1.00**	***0.29***	**1.00**	*0.58*	2.00	*0.63*	**0.19**	***0.12***
	Juniperus virginiana	**0.50**	*0.75*	**1.00**	*0.75*	8.00	3.00	**0.19**	*0.53*	**1.00**	***0.38***	1.50	1.69	1.50	*0.88*	2.00	1.13	**0.13**	***0.14***
	Lavandula angustifolia	**1.00**	*1.00*	4.00	2.00	8.00	5.00	8.00	2.33	1.50	*0.56*	4.00	1.17	1.50	*1.00*	2.00	*0.63*	**0.38**	***0.23***
	Origanum vulgare	**1.00**	*0.56*	2.00	*1.00*	4.00	1.50	6.00	4.00	2.00	*0.58*	4.00	1.17	2.00	1.17	3.00	*0.94*	**0.13**	***0.05***
	Rosmarinus officinalis	**1.00**	*0.63*	4.00	2.00	8.00	3.00	8.00	5.33	2.00	***0.50***	2.00	***0.38***	**1.00**	*0.58*	2.00	*0.63*	**0.13**	***0.27***
	Salvia officinalis	2.00	1.67	4.00	2.00	8.00	3.00	8.00	5.33	1.50	***0.38***	2.00	*0.58*	**0.50**	***0.42***	4.00	1.25	**0.25**	1.03
	Thymus vulgaris	**1.00**	*0.63*	**1.00**	*0.75*	**1.00**	*0.63*	8.00	9.33	**1.00**	***0.25***	**0.50**	*0.56*	**0.75**	***0.44***	2.00	1.13	**0.38**	1.05
	Zingiber officinale	**1.00**	*1.00*	2.00	1.50	8.00	3.00	**0.25**	***0.17***	**1.00**	***0.38***	**0.75**	***0.22***	1.50	*0.69*	8.00	4.50	**0.25**	***0.28***
Lavandula angustifolia (lavender)	*Citrus aurantifolia*	3.00	1.88	2.00	*1.00*	2.00	1.50	2.00	*0.75*	**1.00**	***0.38***	2.00	***0.46***	**1.00**	*0.58*	2.00	*1.00*	**0.25**	***0.29***
	Citrus limon	2.00	1.13	2.00	*1.00*	8.00	6.00	2.00	*0.75*	2.00	*0.75*	2.00	***0.46***	2.00	*1.00*	3.00	2.25	**0.25**	***0.21***
	Citrus sinensis	1.50	*0.84*	2.00	*1.00*	4.00	3.00	2.00	1.25	2.00	*0.75*	2.00	***0.46***	8.00	4.67	2.00	*0.83*	**0.13**	***0.13***
	Commiphora molmol	2.00	1.25	2.00	*1.00*	3.00	3.00	1.50	4.19	**1.00**	*0.75*	2.00	4.33	**1.00**	***0.40***	2.00	*1.00*	**0.50**	*0.75*
	Melaleuca alternifolia	2.00	1.25	**1.00**	*0.58*	2.00	1.50	3.00	1.38	2.00	*0.75*	2.00	*1.00*	4.00	1.83	**1.00**	***0.50***	**0.75**	*0.88*

	Melaleuca viridiflora	2.00	1.25	2.00	*1.00*	2.00	2.00	2.00	1.25	1.50	*0.56*	2.00	***0.46***	8.00	4.67	2.00	*1.00*	**0.19**	***0.47***
	Origanum vulgare	2.00	1.13	**1.00**	***0.50***	2.00	1.50	2.00	1.25	1.50	*0.63*	3.00	*1.00*	1.50	*0.88*	4.00	2.00	**0.25**	***0.19***
	Pimenta racemosa	1.50	2.25	**1.00**	***0.50***	3.00	3.00	**0.75**	*0.84*	**0.75**	***0.38***	2.00	*1.00*	1.50	2.00	1.50	1.13	**0.13**	***0.31***
	Pinus sylvestris	2.00	1.50	2.00	*1.00*	4.00	4.00	1.50	*0.69*	1.50	***0.47***	1.50	***0.34***	8.00	4.67	2.00	*1.00*	**0.19**	*0.84*
	Rosmarinus officinalis	3.00	1.88	2.00	*1.00*	4.00	3.00	3.00	1.88	2.00	*0.75*	3.00	*0.69*	1.50	*0.88*	2.00	*1.00*	**0.19**	***0.47***
	Thymus vulgaris	1.50	*0.94*	**1.00**	*0.75*	**1.00**	*1.00*	2.00	2.25	**1.00**	***0.38***	3.00	3.50	1.50	*0.88*	1.50	1.13	**0.19**	*0.59*
Lavandula burnati (lavandin)	*Citrus limon*	2.00	*0.79*	2.00	*1.00*	4.00	3.00	3.00	2.25	2.00	*0.75*	**0.50**	***0.16***	**1.00**	*0.67*	2.00	1.50	**0.38**	***0.25***
	Citrus sinensis	2.00	*0.79*	2.00	*1.00*	4.00	3.00	3.00	3.00	2.00	*0.75*	2.00	*0.63*	**1.00**	*0.75*	2.00	*0.83*	**0.19**	***0.16***
Lavandula spica (lavender spike)	*Citrus limon*	2.00	1.13	**1.00**	***0.50***	4.00	2.33	**1.00**	***0.42***	8.00	2.00	4.00	*0.75*	**1.00**	***0.50***	2.00	2.00	**0.25**	***0.17***
	Citrus sinensis	2.00	1.13	**1.00**	***0.50***	4.00	2.33	1.50	*1.00*	2.00	***0.50***	4.00	*0.75*	**1.00**	*0.58*	4.00	2.67	**0.25**	***0.21***
Melaleuca cajuputii (cajeput)	*Citrus bergamia*	4.00	*0.75*	**1.00**	***0.50***	2.00	*0.83*	2.00	*0.58*	2.00	***0.50***	1.50	*1.00*	2.00	*0.83*	2.00	*0.63*	**0.25**	1.13
	Citrus limon	2.00	***0.38***	**1.00**	***0.50***	2.00	*0.83*	4.00	1.50	2.00	***0.50***	8.00	4.50	2.00	*0.83*	4.00	3.00	3.00	13.00
	Elettaria cardamonum	2.00	1.25	**1.00**	***0.50***	2.00	*0.83*	2.00	*0.75*	1.50	***0.28***	3.00	2.00	**1.00**	*0.75*	2.00	*1.00*	**0.25**	1.08
	Eucalyptus globulus	2.00	***0.50***	**1.00**	***0.50***	2.00	*1.00*	3.00	1.13	2.00	***0.50***	8.00	5.33	2.00	*1.00*	2.00	*1.00*	2.00	9.00
	Helichrysum italicum	2.00	***0.50***	2.00	*1.00*	2.00	*0.83*	2.00	*0.92*	1.50	*0.56*	6.00	3.75	2.00	*0.67*	3.00	1.75	**0.13**	*1.00*
	Hyssopus officinalis	8.00	3.00	**1.00**	***0.50***	1.50	*0.63*	2.00	1.25	2.00	***0.50***	4.00	2.25	1.50	*0.75*	2.00	*1.00*	2.00	16.00
	Lavandula angustifolia	3.00	1.88	**1.00**	***0.50***	2.00	1.33	2.00	***0.50***	2.00	*0.75*	8.00	5.33	**1.00**	*0.58*	4.00	2.00	**0.25**	1.13
	Melaleuca viridiflora	2.00	***0.50***	**1.00**	***0.50***	2.00	1.33	2.00	1.25	2.00	***0.50***	4.00	2.25	**1.00**	***0.50***	3.00	1.50	**1.00**	6.00
	Myrtus communis	2.00	***0.50***	**1.00**	***0.50***	2.00	*0.83*	2.00	***0.38***	**0.75**	***0.16***	8.00	4.67	2.00	*1.00*	4.00	2.00	**0.25**	1.06
	Pinus sylvestris	2.00	*0.75*	**1.00**	***0.50***	2.00	1.33	2.00	*0.92*	**1.00**	***0.19***	8.00	4.50	4.00	2.00	2.00	*1.00*	6.00	48.00
	Rosa damascena	2.00	*0.75*	**1.00**	***0.50***	2.00	*0.83*	**1.00**	4.13	**1.00**	***0.38***	4.00	4.00	1.50	*0.75*	2.00	*1.00*	**0.50**	2.50
	Rosmarinus officinalis	**1.00**	***0.25***	8.00	4.00	2.00	*0.83*	2.00	1.25	1.50	***0.38***	8.00	4.50	2.00	*1.00*	**0.50**	***0.25***	1.50	9.00
	Santalum austrocaledonicum	**0.50**	***0.40***	**0.13**	***0.28***	**1.00**	***0.42***	**0.25**	2.03	**0.25**	***0.20***	**0.19**	*0.84*	2.00	*0.63*	2.00	4.50	**0.50**	3.33
	Syzygium caryophyllata	**1.00**	***0.38***	**1.00**	*0.75*	2.00	1.33	2.00	*0.75*	2.00	*0.75*	2.00	5.00	2.00	*1.00*	2.00	1.50	**0.19**	2.25

	Thymus vulgaris	3.00	*0.75*	**1.00**	*0.75*	**1.00**	*0.67*	2.00	2.25	**1.00**	***0.25***	**1.00**	1.50	2.00	*1.00*	2.00	1.50	**0.50**	3.33
	Zingiber officinale	2.00	1.25	**0.50**	***0.38***	2.00	*0.83*	1.50	*0.94*	**1.00**	***0.38***	4.00	2.67	**1.00**	***0.38***	3.00	2.25	**0.38**	1.88
Melaleuca viridiflora (niaouli)	*Citrus sinensis*	3.00	*0.56*	2.00	*1.00*	4.00	3.00	2.00	2.00	2.00	***0.50***	8.00	*1.00*	4.00	2.00	**1.00**	***0.42***	**0.38**	*0.94*
	Mentha piperita	2.00	1.25	**1.00**	***0.50***	2.00	1.25	**1.00**	*0.83*	**1.00**	***0.25***	8.00	2.50	4.00	3.00	2.00	*1.00*	**0.50**	1.17
	Pinus sylvestris	2.00	*0.75*	2.00	*1.00*	4.00	4.00	2.00	1.67	2.00	***0.38***	8.00	*1.00*	2.00	*1.00*	2.00	*1.00*	**0.50**	3.00
	Rosmarinus officinalis	3.00	*0.75*	2.00	*1.00*	2.00	1.50	1.50	1.50	2.00	***0.50***	8.00	*1.00*	4.00	2.00	2.00	*1.00*	**0.75**	3.00
	Salvia officinalis	8.00	3.67	2.00	*1.00*	2.00	1.50	**1.00**	*1.00*	2.00	***0.50***	8.00	1.83	8.00	6.00	2.00	*1.00*	**0.50**	3.00
Mentha piperita (peppermint)	*Rosmarinus officinalis*	1.50	*0.94*	**1.00**	***0.50***	3.00	1.13	1.50	1.25	1.50	***0.38***	4.00	1.25	1.50	1.13	2.00	*1.00*	**1.00**	2.33
	Santalum austrocaledonicum	**1.00**	1.17	**0.13**	***0.28***	**0.25**	***0.09***	**0.09**	*0.78*	**0.50**	***0.40***	**0.50**	2.13	**1.00**	*0.56*	2.00	4.50	**0.50**	1.50
	Thymus vulgaris	1.50	*0.94*	**1.00**	*0.75*	**1.00**	*0.63*	**1.00**	1.33	1.50	***0.38***	2.00	2.50	**1.00**	*0.75*	2.00	1.50	**1.00**	3.00
Myrtus communis (myrtle)	*Melaleuca alternifolia*	2.00	***0.50***	**1.00**	*0.58*	**1.00**	***0.50***	2.00	*0.79*	2.00	***0.42***	4.00	1.67	**1.00**	***0.38***	**1.00**	***0.50***	**0.25**	***0.23***
	Mentha piperita	2.00	1.25	**1.00**	***0.50***	2.00	*0.75*	2.00	*0.79*	2.00	***0.42***	6.00	2.00	4.00	3.00	2.00	*1.00*	**0.38**	***0.22***
	Rosmarinus officinalis	2.00	***0.50***	2.00	*1.00*	2.00	*1.00*	4.00	2.25	2.00	***0.42***	8.00	1.17	4.00	2.00	2.00	*1.00*	**0.50**	1.13
	Thymus vulgaris	**1.00**	***0.25***	**0.50**	***0.38***	**1.00**	*0.75*	1.50	1.59	**1.00**	***0.21***	2.00	2.17	3.00	1.50	2.00	1.50	**0.25**	*0.73*
Ocimum basilicum (basil)	*Citrus aurantifolia*	**1.00**	1.13	4.00	3.00	2.00	*1.00*	2.00	1.17	2.00	***0.50***	2.00	***0.29***	2.00	1.50	8.00	6.00	**0.13**	***0.33***
	Citrus bergamia	**1.00**	1.06	4.00	3.00	4.00	2.00	2.00	*1.00*	1.50	***0.38***	3.00	*0.75*	**1.00**	*0.67*	2.00	1.13	**0.13**	***0.31***
	Cymbopogon citratus	**0.50**	*0.75*	2.00	1.50	2.00	1.50	**0.19**	***0.31***	1.50	*0.56*	1.50	*0.63*	**1.00**	*0.56*	4.00	3.00	**0.13**	***0.26***
	Eucalyptus globulus	**1.00**	1.13	4.00	3.00	4.00	2.33	4.00	2.33	2.00	***0.50***	6.00	1.50	1.50	1.13	8.00	6.00	**0.19**	***0.47***
	Hyssopus officinalis	**1.00**	1.25	4.00	3.00	4.00	2.00	1.50	1.25	1.50	***0.38***	1.50	***0.22***	**1.00**	*0.75*	8.00	6.00	**0.75**	4.50
	Juniperus virginiana	**0.50**	*1.00*	**0.50**	***0.50***	2.00	*1.00*	**0.50**	1.50	1.50	*0.56*	2.00	2.17	**1.00**	*0.75*	2.00	2.00	**0.13**	***0.38***
	Lavandula angustifolia	**1.00**	1.50	4.00	3.00	2.00	1.50	2.00	*0.92*	2.00	*0.75*	**1.00**	***0.25***	**1.00**	*0.83*	8.00	6.00	**0.25**	*0.63*
	Melaleuca alternifolia	**1.00**	1.13	2.00	1.67	2.00	*1.00*	**0.63**	***0.42***	**1.00**	***0.25***	**0.75**	***0.31***	1.50	*0.94*	2.00	1.50	**0.13**	***0.33***
	Melaleuca viridiflora	**0.50**	*0.56*	4.00	3.00	2.00	1.50	1.50	1.25	2.00	***0.50***	8.00	1.17	**1.00**	*0.75*	2.00	1.50	**0.25**	*1.00*
	Origanum vulgare	**0.50**	*0.53*	8.00	6.00	2.00	*1.00*	2.00	1.67	2.00	*0.58*	**0.75**	***0.19***	**1.00**	*0.75*	4.00	3.00	**0.13**	***0.28***

	Origanum vulgare	**0.50**	*0.75*	4.00	3.00	4.00	1.50	2.00	1.67	2.00	***0.50***	2.00	***0.50***	**1.00**	*0.75*	4.00	3.00	**0.25**	*0.63*
	Piper nigrum	**1.00**	1.06	8.00	6.00	2.00	*0.83*	**0.75**	***0.38***	2.00	***0.50***	8.00	2.67	**1.00**	*0.75*	4.00	3.00	**0.13**	***0.50***
	Rosmarinus officinalis	**0.50**	*0.56*	4.00	3.00	2.00	*1.00*	1.50	1.25	2.00	***0.50***	8.00	1.17	**1.00**	*0.75*	4.00	3.00	**0.19**	*0.75*
	Salvia officinalis	**1.00**	1.33	4.00	3.00	2.00	*1.00*	**1.00**	*0.83*	1.50	***0.38***	8.00	2.00	1.50	1.50	8.00	6.00	**0.13**	*0.75*
	Santalum austrocaledonicum	**0.50**	*0.83*	**0.13**	***0.31***	2.00	*1.00*	**0.63**	5.21	**0.50**	***0.40***	**0.19**	*0.77*	**0.50**	***0.28***	**0.50**	1.25	**0.38**	1.75
Origanum marjorana (marjoram)	*Citrus sinensis*	2.00	***0.25***	2.00	*1.00*	4.00	2.00	1.50	1.50	2.00	*0.58*	8.00	1.83	8.00	4.00	2.00	*0.83*	**0.13**	***0.09***
	Melaleuca alternifolia	2.00	***0.38***	**1.00**	*0.58*	1.50	*0.75*	2.00	1.67	3.00	*0.88*	4.00	2.00	**1.00**	***0.38***	**1.00**	***0.50***	**0.25**	***0.23***
	Rosmarinus officinalis	2.00	***0.38***	**0.50**	***0.25***	**1.00**	***0.50***	3.00	3.00	2.00	*0.58*	8.00	1.83	8.00	4.00	2.00	*1.00*	**0.13**	***0.28***
	Santalum austrocaledonicum	**0.50**	***0.36***	**0.13**	***0.28***	**0.50**	***0.25***	**0.06**	*0.53*	**1.00**	*0.83*	4.00	16.67	**1.00**	***0.31***	2.00	4.50	**0.13**	***0.36***
Origanum vulgare (origanum)	*Melaleuca alternifolia*	2.00	1.25	**1.00**	*0.58*	**1.00**	***0.38***	2.00	1.67	2.00	***0.50***	4.00	2.00	**1.00**	***0.38***	3.00	1.50	**0.50**	*0.58*
	Pinus sylvestris	2.00	1.50	2.00	*1.00*	2.00	1.25	2.00	1.67	**1.00**	***0.19***	8.00	1.83	2.00	*1.00*	2.00	*1.00*	**0.50**	2.25
	Rosmarinus officinalis	2.00	1.25	2.00	*1.00*	2.00	*0.75*	2.00	2.00	**1.00**	***0.25***	8.00	1.83	**1.00**	***0.50***	3.00	1.50	**0.50**	1.25
Pinus sylvestris (pine)	*Melaleuca alternifolia*	**1.00**	***0.38***	**1.00**	*0.58*	**0.50**	***0.38***	2.00	1.33	**1.00**	***0.19***	**0.75**	***0.30***	2.00	*0.75*	2.00	*1.00*	**0.25**	1.17
	Pimenta racemosa	**1.00**	1.25	**1.00**	***0.50***	2.00	2.00	1.50	2.00	**1.00**	***0.31***	4.00	1.58	**0.75**	*0.94*	**1.00**	*0.75*	**0.50**	3.00
	Rosmarinus officinalis	2.00	*0.75*	2.00	*1.00*	4.00	3.00	3.00	2.50	4.00	*0.75*	8.00	*1.00*	8.00	4.00	2.00	*1.00*	**0.25**	1.50
	Santalum austrocaledonicum	**1.00**	*0.92*	**0.13**	***0.28***	**0.50**	***0.38***	**0.25**	2.08	**0.50**	***0.36***	**0.50**	2.03	**1.00**	***0.31***	2.00	4.50	**0.50**	3.33
	Thymus vulgaris	**1.00**	***0.38***	**0.50**	***0.38***	**0.75**	*0.75*	2.00	2.67	1.50	***0.28***	2.00	2.13	**1.00**	***0.50***	2.00	1.50	**0.50**	3.33
Piper nigrum (black pepper)	*Citrus aurantifolia*	2.00	***0.38***	2.00	*1.00*	2.00	*0.83*	8.00	3.33	2.00	***0.50***	3.00	*0.94*	2.00	*1.00*	3.00	1.50	4.00	10.67
	Citrus bergamia	8.00	*1.00*	**1.00**	***0.50***	2.00	*0.83*	4.00	1.33	**1.00**	***0.25***	8.00	3.33	2.00	*0.83*	2.00	*0.63*	**0.25**	*0.63*
	Citrus limon	8.00	*1.00*	2.00	*1.00*	2.00	*0.83*	6.00	2.50	**1.00**	***0.25***	3.00	*0.94*	3.00	1.25	4.00	3.00	**0.19**	***0.44***
	Citrus sinensis	2.00	***0.25***	2.00	*1.00*	2.00	*0.83*	**0.38**	***0.25***	2.00	***0.50***	8.00	2.50	3.00	1.50	3.00	1.25	1.50	3.75
	Cupressus sempervirens	4.00	*0.92*	2.00	*1.00*	2.00	***0.46***	8.00	2.67	**1.00**	***0.25***	1.50	*0.63*	2.00	*1.00*	3.00	*0.94*	**0.25**	*0.75*
	Juniperus virginiana	3.00	3.19	**0.50**	***0.38***	**1.00**	***0.42***	**0.19**	*0.53*	**1.00**	***0.38***	**1.00**	1.25	**1.00**	***0.50***	2.00	1.50	**1.00**	3.00
	Lavandula angustifolia	2.00	1.13	2.00	*1.00*	2.00	1.33	8.00	2.33	**1.00**	***0.38***	3.00	1.25	**1.00**	*0.58*	3.00	1.50	**0.25**	*0.63*

	Origanum vulgare	8.00	*1.00*	**1.00**	***0.50***	2.00	*0.83*	2.00	1.33	2.00	*0.58*	8.00	3.33	2.00	*1.00*	2.00	*1.00*	2.00	4.50
	Rosmarinus officinalis	4.00	*0.75*	2.00	*1.00*	2.00	*0.83*	2.00	1.33	2.00	***0.50***	8.00	2.50	2.00	*1.00*	**0.75**	***0.38***	**0.13**	***0.50***
	Santalum austrocaledonicum	**0.50**	***0.36***	**0.25**	*0.56*	**0.13**	***0.05***	**0.25**	2.04	**0.38**	***0.30***	**0.38**	1.59	2.00	*0.63*	2.00	4.50	**0.25**	1.17
	Styrax benzoin	4.00	*0.75*	2.00	*1.00*	2.00	*0.58*	**0.38**	***0.19***	2.00	***0.50***	6.00	3.00	**1.00**	***0.50***	4.00	2.00	**0.50**	1.67
	Thymus vulgaris	2.00	***0.38***	**0.50**	***0.38***	**1.00**	*0.67*	**1.00**	1.17	4.00	*1.00*	**1.00**	1.25	**1.00**	***0.50***	3.00	2.25	**1.00**	4.67
Rosa damascena (rose)	*Santalum austrocaledonicum*	**0.25**	***0.23***	**0.25**	*0.56*	**1.00**	***0.50***	**0.06**	*0.75*	**0.25**	***0.23***	**0.75**	3.38	3.00	*0.94*	**1.00**	2.25	**0.13**	***0.46***
Rosmarinus officinalis (rosemary)	*Melaleuca alternifolia*	2.00	***0.50***	**0.75**	***0.44***	**1.00**	***0.50***	3.00	2.50	8.00	2.00	1.50	*0.59*	3.00	1.13	**1.00**	***0.50***	**0.09**	***0.25***
Salvia officinalis (sage)	*Melaleuca alternifolia*	3.00	1.38	**1.00**	*0.58*	**1.00**	***0.50***	4.00	3.33	**1.00**	***0.25***	3.00	1.50	**1.00**	*0.63*	**1.00**	***0.50***	**0.50**	2.33
	Santalum austrocaledonicum	**0.38**	***0.38***	**0.13**	***0.28***	**1.00**	***0.50***	**0.13**	1.06	**0.50**	***0.40***	4.00	16.67	**1.00**	*0.56*	**1.00**	2.25	**0.50**	3.33
	Thymus vulgaris	2.00	*0.92*	**1.00**	*0.75*	**1.00**	*0.75*	**1.00**	1.50	**1.00**	***0.25***	2.00	2.33	**1.00**	*0.75*	**1.00**	*0.75*	**0.75**	5.00
Salvia sclarea (clary sage)	*Boswellia carterii*	4.00	***0.50***	**1.00**	***0.50***	4.00	2.25	8.00	3.33	1.50	***0.44***	2.00	2.33	8.00	2.50	2.00	*0.63*	**0.19**	*0.84*
	Citrus aurantifolia	4.00	*0.75*	2.00	*1.00*	2.00	*0.63*	2.00	*0.83*	1.50	***0.38***	1.50	***0.34***	8.00	2.50	2.00	*0.63*	**0.19**	*0.88*
	Citrus bergamia	8.00	*1.00*	**1.00**	***0.50***	4.00	1.25	8.00	2.67	1.50	***0.38***	1.50	***0.50***	8.00	1.83	2.00	***0.25***	**0.13**	*0.56*
	Juniperus virginiana	2.00	2.13	**1.00**	*0.75*	2.00	*0.63*	3.00	8.50	**1.00**	***0.38***	**0.50**	*0.58*	8.00	2.50	2.00	1.13	**0.13**	*0.63*
	Lavandula angustifolia	**1.00**	*0.56*	**1.00**	***0.50***	2.00	1.13	8.00	2.33	2.00	*0.75*	1.50	***0.50***	2.00	*0.79*	2.00	*0.63*	**0.50**	2.25
	Lavandula burnati	**1.00**	***0.40***	2.00	*1.00*	2.00	1.13	2.00	1.33	2.00	*0.75*	2.00	*0.83*	2.00	1.13	2.00	*0.63*	**0.19**	*0.81*
	Rosa damascena	**1.00**	***0.31***	**1.00**	***0.50***	2.00	*0.63*	**1.00**	4.17	**1.00**	***0.38***	1.50	*1.00*	4.00	1.25	2.00	*0.63*	**0.13**	*0.63*
	Tagetes minuta	**1.00**	*0.56*	2.00	*1.00*	2.00	***0.38***	2.00	2.33	**1.00**	***0.38***	2.00	*0.67*	2.00	1.13	2.00	*0.63*	**0.50**	2.25
Santalum austrocaledonicum (sandalwood)	*Melaleuca alternifolia*	**1.00**	*0.79*	**0.25**	*0.58*	**0.50**	***0.25***	**0.50**	4.17	**0.38**	***0.30***	1.50	6.50	**1.00**	***0.19***	**1.00**	2.25	**0.25**	*0.83*
Styrax benzoin (benzoin)	*Boswellia carterii*	4.00	*0.75*	2.00	*1.00*	2.00	1.25	8.00	4.67	2.00	*0.58*	2.00	2.50	2.00	*1.00*	2.00	*1.00*	**0.75**	1.38
	Citrus bergamia	8.00	1.50	2.00	*1.00*	8.00	3.00	8.00	4.00	1.50	***0.38***	1.50	*0.63*	8.00	3.33	2.00	*0.63*	**0.75**	1.38
	Citrus limon	4.00	*0.75*	2.00	*1.00*	8.00	3.00	**1.00**	*0.58*	1.50	***0.38***	8.00	2.50	2.00	*0.83*	2.00	1.50	**1.00**	1.67
	Citrus sinensis	8.00	1.50	3.00	1.50	4.00	1.50	3.00	2.50	2.00	***0.50***	8.00	2.50	2.00	*1.00*	4.00	1.67	**0.75**	1.38

	Commiphora molmol	4.00	*1.00*	2.00	*1.00*	2.00	1.25	**0.13**	***0.38***	**1.00**	*0.63*	3.00	6.75	8.00	2.50	2.00	*1.00*	**0.50**	1.17
	Coriandrum sativum	**0.25**	***0.28***	8.00	6.00	**1.00**	*0.63*	**0.75**	1.75	**1.00**	***0.38***	**1.00**	1.58	1.50	1.88	2.00	1.17	**0.06**	1.08
	Cupressus sempervirens	6.00	1.75	2.00	*1.00*	2.00	***0.38***	4.00	2.00	1.50	***0.38***	3.00	1.25	2.00	*1.00*	2.00	0.63	**0.75**	1.75
	Lavandula angustifolia	**1.00**	*0.63*	2.00	*1.00*	4.00	2.50	**1.00**	***0.46***	2.00	*0.75*	8.00	3.33	**1.00**	*0.58*	4.00	2.00	**0.13**	***0.23***
	Mentha piperita	**1.00**	*0.63*	2.00	*1.00*	2.00	***0.50***	**1.00**	0.67	**1.00**	***0.25***	2.00	*1.00*	**1.00**	*0.75*	4.00	2.00	**0.13**	***0.21***
	Rosa damascena	2.00	*0.75*	**1.00**	***0.50***	**1.00**	***0.38***	**0.50**	2.17	1.50	*0.56*	**0.50**	***0.38***	2.00	*1.00*	**1.00**	***0.50***	**0.50**	1.17
	Santalum austrocaledonicum	**1.00**	*0.79*	**0.13**	***0.28***	**0.50**	***0.19***	**0.63**	5.21	**0.38**	***0.30***	**0.38**	1.59	8.00	2.50	**0.75**	1.69	8.00	32.00
Syzygium caryophyllata (clove)	*Cinnamonum zeylanicum*	**0.25**	***0.19***	**0.50**	*0.75*	**1.00**	*0.75*	**0.75**	1.69	**0.50**	***0.25***	**0.75**	2.00	**0.50**	***0.38***	2.00	3.00	**0.13**	1.08
	Citrus bergamia	**1.00**	***0.31***	**1.00**	*0.75*	2.00	1.50	1.50	*0.63*	**1.00**	***0.38***	2.00	4.33	**1.00**	***0.42***	2.00	1.13	**0.13**	1.06
	Citrus limon	**1.00**	***0.31***	**1.00**	*0.75*	2.00	1.50	3.00	1.50	**1.00**	***0.38***	**1.00**	2.06	3.00	1.25	2.00	2.00	**0.13**	1.04
	Citrus sinensis	**1.00**	***0.31***	2.00	1.50	2.00	1.50	8.00	6.00	**1.00**	***0.38***	**1.00**	2.06	2.00	*1.00*	2.00	1.33	**0.50**	4.25
	Commiphora molmol	**0.50**	***0.19***	**1.00**	*0.75*	2.00	2.00	8.00	23.33	**0.50**	***0.38***	**0.75**	3.00	2.00	*0.63*	2.00	1.50	**0.06**	*0.56*
	Cymbopogon citratus	**1.00**	*0.75*	**0.50**	***0.38***	**1.00**	*1.00*	1.50	2.38	**0.75**	***0.38***	**0.50**	1.17	**0.50**	***0.16***	2.00	1.50	**0.25**	2.02
	Eucalyptus globulus	**1.00**	***0.38***	**1.00**	*0.75*	2.00	1.67	2.00	*1.00*	**1.00**	***0.38***	**0.75**	1.63	**0.50**	***0.25***	2.00	1.50	**0.50**	4.25
	Juniperus virginiana	**0.50**	*0.63*	**0.50**	***0.50***	**1.00**	*0.75*	**0.19**	*0.55*	**1.00**	***0.50***	**0.38**	1.13	**1.00**	***0.50***	2.00	2.00	**0.13**	1.13
	Lavandula angustifolia	**1.00**	*0.75*	**1.00**	*0.75*	2.00	2.00	1.50	*0.56*	**1.00**	***0.50***	**0.75**	1.63	**1.00**	*0.58*	2.00	1.50	**0.13**	1.06
	Melaleuca alternifolia	**1.00**	***0.38***	**1.00**	*0.83*	**1.00**	*0.75*	**0.50**	***0.29***	**1.00**	***0.38***	**0.50**	1.17	**1.00**	***0.38***	2.00	1.50	**0.06**	*0.54*
	Mentha piperita	**1.00**	*0.75*	**1.00**	*0.75*	**1.00**	*0.63*	6.00	3.50	**1.00**	***0.38***	**1.00**	2.25	**1.00**	*0.75*	2.00	1.50	**0.25**	2.08
	Ocimum basilicum	**0.50**	*0.63*	**1.00**	*1.00*	2.00	1.50	**1.00**	*0.58*	**1.00**	***0.38***	1.50	3.13	**0.50**	***0.38***	2.00	2.00	**0.13**	1.25
	Pinus sylvestris	**1.00**	***0.50***	**1.00**	*0.75*	2.00	2.00	8.00	4.67	**1.00**	***0.31***	**1.00**	2.06	2.00	*1.00*	2.00	1.50	**0.06**	*0.75*
	Piper nigrum	**1.00**	***0.31***	**1.00**	*0.75*	2.00	1.33	1.50	*0.63*	**1.00**	***0.38***	**1.00**	2.25	4.00	2.00	2.00	1.50	**0.13**	1.25
	Rosmarinus officinalis	**1.00**	***0.38***	**1.00**	*0.75*	2.00	1.50	4.00	3.00	**1.00**	***0.38***	**1.00**	2.06	2.00	*1.00*	2.00	1.50	**0.09**	*0.94*
	Styrax benzoin	**1.00**	***0.38***	**0.50**	***0.38***	2.00	1.25	**0.75**	***0.44***	4.00	1.50	1.50	3.38	4.00	2.00	2.00	1.50	8.00	74.67
	Thymus vulgaris	**1.00**	***0.38***	**0.50**	***0.50***	**0.50**	***0.50***	**1.00**	1.25	4.00	1.50	**0.50**	1.50	**0.50**	***0.25***	2.00	2.00	**0.13**	1.33

	Zingiber officinale	**1.00**	*0.75*	**0.50**	***0.50***	**1.00**	*0.75*	1.50	1.13	**0.50**	***0.25***	**0.50**	1.08	**1.00**	***0.38***	2.00	2.00	**0.25**	2.25
Thymus vulgaris (thyme)	*Melaleuca alternifolia*	2.00	***0.50***	**0.50**	***0.42***	**1.00**	*0.75*	2.00	2.67	2.00	***0.50***	1.50	2.00	**1.00**	***0.38***	2.00	1.50	**0.09**	***0.31***
Zingiber officinale (ginger)	*Citrus limon*	6.00	3.38	2.00	1.50	4.00	2.00	**0.50**	***0.50***	2.00	*0.75*	4.00	*0.92*	**1.00**	***0.38***	4.00	2.67	**0.75**	1.13
	Citrus sinensis	2.00	1.25	**1.00**	*0.75*	4.00	3.00	**0.13**	***0.40***	**1.00**	*0.75*	2.00	4.33	**1.00**	***0.19***	4.00	3.00	**0.38**	*0.75*
	Commiphora molmol	**1.00**	*1.00*	**1.00**	*0.75*	2.00	1.50	**0.19**	***0.34***	4.00	2.00	3.00	1.50	**0.50**	***0.09***	4.00	3.00	**0.25**	***0.27***
	Cymbopogon citratus	2.00	1.25	**1.00**	*0.83*	1.50	*0.75*	**1.00**	*0.83*	**1.00**	***0.38***	4.00	2.00	**0.75**	***0.19***	4.00	3.00	**0.50**	*0.83*
	Melaleuca alternifolia	2.00	2.00	2.00	1.50	2.00	*0.75*	**0.50**	***0.42***	**1.00**	***0.38***	4.00	1.67	**0.75**	***0.47***	4.00	3.00	**0.38**	***0.50***
	Mentha piperita	1.50	2.25	**1.00**	*0.75*	2.00	1.50	**0.13**	***0.19***	**1.00**	***0.50***	4.00	2.00	**1.00**	1.13	4.00	4.00	**0.38**	1.13
	Pimenta racemosa	2.00	1.25	2.00	1.50	2.00	*1.00*	**0.38**	***0.38***	**1.00**	***0.38***	8.00	1.83	2.00	*0.75*	4.00	3.00	**0.50**	1.50
	Rosmarinus officinalis	2.00	1.67	2.00	1.50	2.00	*1.00*	**0.19**	***0.19***	**0.75**	***0.28***	4.00	1.33	**0.50**	***0.31***	4.00	3.00	**0.50**	2.50
	Salvia officinalis	**0.50**	*0.58*	**0.13**	***0.31***	**0.25**	***0.13***	**0.62**	5.30	2.00	1.83	**0.13**	*0.52*	**0.75**	***0.14***	4.00	10.00	**0.25**	*0.92*
	Santalum austrocaledonicum	**1.00**	*0.63*	**1.00**	*1.00*	1.50	1.13	**1.00**	1.50	**1.00**	***0.38***	2.00	2.33	**0.50**	***0.19***	4.00	4.00	**0.50**	1.83
	Thymus vulgaris	2.00	*0.63*	2.00	*1.00*	4.00	4.00	3.00	1.25	2.00	*0.83*	4.00	5.33	**0.75**	*0.56*	2.00	*1.00*	**0.25**	***0.16***
Standard error mean		0.08		0.08		0.08		0.13		0.06		0.13		0.11		0.06		0.05	
Positive control (ciprofloxacin)		0.50 x 10^{-3}		0.50 x 10^{-3}		0.50 x 10^{-3}		0.50 x 10^{-3}		0.50 x 10^{-3}		0.25 x 10^{-3}		1.00 x 10^{-3}		0.50 x 10^{-3}		n.a.	
Positive control (amphotericin b)		n.a.		n.a.		n.a.		n.a.		n.a.		n.a.		n.a.		n.a.		0.50 x 10^{-3}	
Negative control (acetone in water)		> 8.00		> 8.00		> 8.00		> 8.00		> 8.00		> 8.00		> 8.00		> 8.00		> 8.00	

MIC* denotes noteworthy antimicrobial effect (MIC less than or equal to 1 mg/mL); **ΣFIC**** in bold and italics denotes synergistic antimicrobial effect (ΣFIC less than or equal to 0.50); while ΣFIC in italics only denotes additive antimicrobial effect (ΣFIC greater than 0.50 and less than or equal to 1.00).

Table S2. The mean percentage brine-shrimp viability after 48 hours (n = 3) and ΣFIC values of the essential oil combinations investigated

Essential oil combinations		Individual brine-shrimp mortality (%)*		Combined brine-shrimp mortality (%)*	ΣFIC**
**Essential oil 1**	**Essential oil 2**	**Essential oil 1**	**Essential oil 2**		
Abies balsamea (fir)	*Boswellia carterii*	**0.69**	**1.39**	81.94	88.92
	Carum carvi		96.32	90.61	66.23
	Juniperus virginiana		**0.57**	77.71	124.01
	Lavandula angustifolia		**9.59**	59.61	46.37
	Melaleuca viridiflora		**4.14**	78.16	66.16
	Myrtus communis		**0.00**	78.02	3957.84
	Ocimum basilicum		**13.04**	91.81	70.15
Amyris balsamifera (amyris)	*Boswellia carterii*	**1.40**	**1.39**	**6.14**	4.40
	Canarium luzonicum		**0.73**	98.55	102.90
	Ferula galbaniflua		**15.77**	75.82	29.52
	Lavandula angustifolia		**9.59**	**13.31**	5.46
	Salvia officinalis		**0.82**	**45.43**	44.05
	Styrax benzoin		**2.47**	**5.65**	3.16
Boswellia carterii (frankincense)	*Cinnamonum zeylanicum*	**1.39**	98.49	**11.38**	4.15
	Citrus bergamia		**1.22**	81.61	62.81
	Citrus limon		**1.75**	**2.38**	1.54
	Citrus sinensis		**0.96**	**8.95**	7.90
	Commiphora molmol		**1.03**	**6.77**	5.73
	Elettaria cardamonum		**0.00**	**25.69**	1293.67
	Ferula galbaniflua		**15.77**	**17.50**	6.84
	Helichrysum italicum		**9.50**	**41.38**	17.05
	Juniperus virginiana		**0.57**	65.06	79.99
	Lavandula angustifolia		**9.59**	**3.30**	1.36
	Melaleuca viridiflora		**4.14**	62.54	30.03
	Ocimum basilicum		**13.04**	83.62	33.25
	Origanum vulgare		99.67	**1.39**	***0.51***
	Pimenta racemosa		100.00	91.41	33.31
	Pinus sylvestris		**1.04**	**2.37**	1.99
	Piper nigrum		**1.32**	88.62	65.42
	Rosmarinus officinalis		**0.71**	**2.75**	2.93
	Salvia officinalis		**0.82**	**3.75**	3.64
	Santalum austrocaledonicum		99.22	**10.07**	3.65
	Zingiber officinale		**0.19**	78.57	233.30
Canarium luzonicum (elemi)	*Boswellia carterii*	**0.73**	**1.39**	88.42	92.47
	Citrus sinensis		**0.96**	**4.03**	4.88
	Elettaria cardamonum		**0.00**	74.32	3767.22
	Lavandula angustifolia		**9.59**	**7.85**	5.80

Essential oil combinations		Individual brine-shrimp mortality (%)*		Combined brine-shrimp mortality (%)*	ΣFIC**
**Essential oil 1**	**Essential oil 2**	**Essential oil 1**	**Essential oil 2**		
	Rosmarinus officinalis		**0.71**	84.18	117.35
	Zingiber officinale		**0.19**	**3.32**	10.95
Carum carvi (caraway)	*Boswellia carterii*	96.32	**1.39**	93.68	34.15
	Canarium luzonicum		**0.73**	96.40	66.68
	Citrus sinensis		**0.96**	61.69	32.62
	Coriandrum sativum		**11.95**	92.15	4.33
	Elettaria cardamonum		**0.00**	84.81	4241.13
	Ferula galbaniflua		**15.77**	95.00	3.50
	Laurus nobilis		**3.04**	82.32	13.98
	Lavandula angustifolia		**9.59**	91.05	5.22
	Ocimum basilicum		**13.04**	88.58	3.86
	Styrax benzoin		**2.47**	69.30	14.38
	Zingiber officinale		**0.19**	95.87	250.71
Cinnamonum camphora (camphor)	*Citrus limon*		**1.75**	80.23	51.20
	Lavandula angustifolia		**9.59**	81.82	33.11
	Melaleuca cajuputii		**0.60**	70.64	84.10
	Myrtus communis		**0.00**	85.26	4292.87
	Ocimum basilicum		**13.04**	70.06	27.36
	Pinus sylvestris		**1.04**	86.11	71.81
	Zingiber officinale		**0.19**	66.21	196.16
Cinnamonum zeylanicum (cinnamon)	*Citrus limon*	**1.42**	**1.75**	80.28	23.34
	Citrus sinensis		**0.96**	83.31	44.04
	Commiphora molmol		**1.03**	81.53	40.08
	Elettaria cardamonum		**0.00**	67.24	3362.11
	Eucalyptus globulus		**4.21**	82.24	10.19
	Ferula galbaniflua		**15.77**	90.78	3.34
	Lavandula angustifolia		**9.59**	81.03	4.64
	Melaleuca alternifolia		**1.07**	84.98	39.97
	Ocimum basilicum		**13.04**	83.60	3.63
	Pinus sylvestris		**1.04**	86.19	41.93
	Rosmarinus officinalis		**0.71**	82.40	58.72
	Styrax benzoin		**2.47**	76.69	15.91
	Thymus vulgaris		97.76	88.63	*0.90*
	Zingiber officinale		**0.19**	90.60	236.94
Citrus aurantifolia (lime)	*Rosa damascena*	**0.36**	97.32	**5.71**	7.90
	Rosmarinus officinalis		**0.71**	**0.00**	***0.00***
	Salvia officinalis		**0.82**	**2.09**	4.16
Citrus bergamia (bergamot)	*Citrus aurantifolia*	**1.22**	**0.36**	65.27	116.85
	Citrus limon		**1.75**	71.51	49.76

Essential oil combinations		Individual brine-shrimp mortality (%)*		Combined brine-shrimp mortality (%)*	ΣFIC**
**Essential oil 1**	**Essential oil 2**	**Essential oil 1**	**Essential oil 2**		
	Commiphora molmol		**1.03**	94.88	85.09
	Coriandrum sativum		**11.95**	83.19	37.61
	Cupressus sempervirens		**1.02**	**0.65**	*0.59*
	Eucalyptus globulus		**4.21**	**9.02**	4.77
	Helichrysum italicum		**9.50**	67.87	31.41
	Lavandula angustifolia		**9.59**	78.09	36.11
	Lavandula burnati		**0.16**	97.20	351.89
	Lavandula spica		**1.01**	80.68	73.07
	Melaleuca alternifolia		**1.07**	95.54	83.64
	Myrtus communis		**0.00**	92.06	4640.95
	Origanum vulgare		99.67	90.53	37.59
	Pinus sylvestris		**1.04**	67.39	60.09
	Rosa damascena		97.32	92.23	38.31
	Rosmarinus officinalis		**0.71**	98.44	110.04
	Salvia officinalis		**0.82**	74.63	76.27
	Santalum austrocaledonicum		99.22	95.87	39.81
	Thymus vulgaris		97.76	88.66	36.83
Citrus limon (lemon)	*Citrus sinensis*	**1.75**	**0.96**	**6.74**	5.46
	Commiphora molmol		**1.03**	**6.40**	4.94
	Melaleuca viridiflora		**4.14**	**11.69**	4.75
	Mentha piperita		**6.98**	**4.29**	1.53
	Pimenta racemosa		100.00	**2.95**	*0.86*
	Rosa damascena		97.32	66.14	19.23
	Santalum austrocaledonicum		99.22	**4.00**	1.16
	Thymus vulgaris		97.76	**5.27**	1.53
Citrus sinensis (orange)	*Melaleuca alternifolia*	**0.96**	**1.07**	**37.66**	37.24
	Pimenta racemosa		100.00	88.57	46.81
	Rosa damascena		97.32	87.30	46.15
	Rosmarinus officinalis		**0.71**	**4.35**	5.35
	Salvia officinalis		**0.82**	**42.21**	47.92
	Thymus vulgaris		97.76	84.07	44.44
Commiphora molmol (myrrh)	*Mentha piperita*	**1.03**	**6.98**	**2.17**	1.21
	Pinus sylvestris		**1.04**	57.52	55.67
	Santalum austrocaledonicum		99.22	59.32	29.16
	Thymus vulgaris		97.76	74.26	36.51
Coriandrum sativum (coriander)	*Boswellia carterii*	**11.95**	**1.39**	**11.17**	4.48
	Citrus limon		**1.75**	**4.98**	1.63
	Citrus sinensis		**0.96**	**15.78**	8.92
	Cupressus sempervirens		**1.02**	71.60	38.17

Essential oil combinations		Individual brine-shrimp mortality (%)*		Combined brine-shrimp mortality (%)*	ΣFIC**
Essential oil 1	Essential oil 2	Essential oil 1	Essential oil 2		
	Cymbopogon citratus		100.00	66.36	3.11
	Ferula galbaniflua		**15.77**	**7.31**	*0.54*
	Melaleuca viridiflora		**4.14**	**5.63**	*0.91*
	Myrtus communis		**0.00**	85.13	4259.86
	Pinus sylvestris		**1.04**	**7.25**	3.79
	Piper nigrum		**1.32**	87.03	36.61
	Salvia officinalis		**0.82**	**3.90**	2.55
	Santalum austrocaledonicum		99.22	**41.79**	1.96
	Zingiber officinale		**0.19**	**6.98**	18.52
Cupressus sempervirens (cypress)	*Boswellia carterii*	**1.02**	**1.39**	56.73	48.26
	Citrus limon		**1.75**	60.20	46.77
	Citrus sinensis		**0.96**	79.97	81.15
	Commiphora molmol		**1.03**	**7.10**	6.95
	Hyssopus officinalis		**6.65**	**8.80**	4.99
	Juniperus virginiana		**0.57**	**10.36**	14.11
	Lavandula angustifolia		**9.59**	**39.18**	21.29
	Melaleuca alternifolia		**1.07**	**8.87**	8.48
	Mentha piperita		**6.98**	72.09	40.58
	Origanum vulgare		99.67	83.11	41.24
	Pinus sylvestris		**1.04**	**3.61**	3.51
	Rosmarinus officinalis		**0.71**	93.02	111.51
	Salvia officinalis		**0.82**	**17.25**	19.03
	Salvia sclarea		**0.20**	**11.81**	34.67
	Santalum austrocaledonicum		99.22	86.66	43.01
Cymbopogon citratus (lemongrass)	*Citrus sinensis*	100.00	**0.96**	87.00	45.98
	Melaleuca alternifolia		**1.07**	89.12	41.91
	Melaleuca viridiflora		**4.14**	73.30	9.21
	Mentha piperita		**6.98**	80.89	6.20
	Pimenta racemosa		100.00	81.43	*0.81*
	Rosmarinus officinalis		**0.71**	79.81	56.87
	Santalum austrocaledonicum		99.22	80.83	*0.81*
	Thymus vulgaris		97.76	82.01	*0.83*
Daucus carota (carrot seed)	*Citrus aurantifolia*	**11.19**	**0.36**	89.19	127.07
	Citrus bergamia		**1.22**	90.49	41.16
	Citrus limon		**1.75**	87.79	29.00
	Citrus sinensis		**0.96**	86.48	49.14
	Lavandula angustifolia		**9.59**	86.07	8.34
	Rosmarinus officinalis		**0.71**	92.73	69.76
	Citrus limon	**0.00**	**1.75**	91.49	4600.85

Essential oil combinations		Individual brine-shrimp mortality (%)*		Combined brine-shrimp mortality (%)*	ΣFIC**
**Essential oil 1**	**Essential oil 2**	**Essential oil 1**	**Essential oil 2**		
	Coriandrum sativum		**11.95**	**35.07**	1754.74
Elettaria cardamonum (cardamom)	*Myrtus communis*		**0.00**	66.43	6642.89
	Pinus sylvestris		**1.04**	95.25	4808.41
	Citrus limon		**1.75**	68.65	27.76
	Commiphora molmol		**1.03**	80.22	48.56
	Coriandrum sativum		**11.95**	**45.52**	7.31
	Cupressus sempervirens		**1.02**	**34.17**	20.85
	Cymbopogon citratus		100.00	86.66	10.73
	Juniperus virginiana		**0.57**	83.87	82.95
	Lavandula angustifolia		**9.59**	**34.08**	5.83
Eucalyptus globulus (eucalyptus)	*Melaleuca alternifolia*		**1.07**	79.10	46.20
	Mentha piperita		**6.98**	78.32	14.92
	Origanum vulgare		99.67	74.69	9.25
	Pinus sylvestris		**1.04**	83.41	50.06
	Rosmarinus officinalis		**0.71**	86.89	71.81
	Styrax benzoin	**4.21**	**2.47**	**8.52**	2.74
	Thymus vulgaris		97.76	78.27	9.70
	Zingiber officinale		**0.19**	55.97	152.72
	Canarium luzonicum		**0.73**	**4.19**	3.01
	Commiphora molmol		**1.03**	**3.86**	2.00
	Elettaria cardamonum		**0.00**	**5.79**	289.90
	Melaleuca viridiflora		**4.14**	**2.69**	***0.41***
Ferula galbaniflua (galbanum)	*Pimenta racemosa*		100.00	92.18	3.38
	Pinus sylvestris		**1.04**	**8.19**	4.20
	Rosa damascena		97.32	**4.07**	***0.15***
	Tagetes minuta		**1.30**	**3.98**	1.66
	Zingiber officinale		**0.19**	**28.08**	74.18
	Citrus limon		**1.75**	81.46	23.75
	Hyssopus officinalis		**6.65**	72.32	5.87
	Lavandula angustifolia		**9.59**	80.29	4.66
	Melaleuca viridiflora		**4.14**	82.73	10.48
Foeniculum vulgare (fennel)	*Ocimum basilicum*	84.26	**13.04**	82.97	3.67
	Origanum vulgare		99.67	91.49	1.00
	Rosa damascena		97.32	70.62	*0.78*
	Rosmarinus officinalis		**0.71**	84.54	60.32
	Santalum austrocaledonicum		99.22	83.87	*0.92*
	Citrus sinensis		**0.96**	77.35	44.56
Helichrysum italicum (immortelle)	*Lavandula angustifolia*	**9.50**	**9.59**	**13.45**	1.41
	Lavandula burnati		**0.16**	77.22	251.94

Essential oil combinations		Individual brine-shrimp mortality (%)*		Combined brine-shrimp mortality (%)*	ΣFIC**
**Essential oil 1**	**Essential oil 2**	**Essential oil 1**	**Essential oil 2**		
	Lavandula spica		**1.01**	**26.38**	14.46
	Rosa damascena		97.32	**7.59**	***0.44***
Hyssopus officinalis (hyssop)	*Citrus limon*	**6.65**	**1.75**	**8.59**	3.10
	Citrus sinensis		**0.96**	**7.88**	4.72
	Lavandula angustifolia		**9.59**	**4.87**	*0.62*
	Rosmarinus officinalis		**0.71**	**13.07**	10.23
	Salvia officinalis		**0.82**	**7.10**	4.88
	Santalum austrocaledonicum		99.22	**32.10**	2.58
Illicium verum (star-anise)	*Carum carvi*		96.32	76.86	2.88
	Coriandrum sativum		**11.95**	87.02	6.45
	Cupressus sempervirens		**1.02**	84.35	44.17
	Elettaria cardamonum		**0.00**	81.04	4054.85
	Foeniculum vulgare		84.26	87.37	3.34
Juniperus virginiana (cederwood)	*Cinnamonum zeylanicum*	**15.47**	98.49	91.95	80.47
	Citrus bergamia		**1.22**	91.07	116.60
	Citrus limon		**1.75**	88.74	102.56
	Commiphora molmol		**1.03**	82.90	112.47
	Cymbopogon citratus		100.00	91.00	79.64
	Lavandula angustifolia		**9.59**	87.56	80.76
	Mentha piperita		**6.98**	85.40	80.43
	Origanum vulgare		99.67	86.16	75.41
	Pinus sylvestris		**1.04**	57.46	77.66
	Rosa damascena		97.32	72.90	63.81
	Rosmarinus officinalis		**0.71**	79.57	125.53
	Santalum austrocaledonicum		99.22	83.54	73.11
	Styrax benzoin		**2.47**	93.60	100.38
	Thymus vulgaris		97.76	68.89	60.30
	Zingiber officinale		**0.19**	82.73	287.92
Laurus nobilis (bay)	*Citrus limon*	**3.04**	**1.75**	**1.57**	*0.71*
	Citrus sinensis		**0.96**	**30.80**	21.20
	Coriandrum sativum		**11.95**	72.42	14.95
	Eucalyptus globulus		**4.21**	**17.86**	5.06
	Juniperus virginiana		**0.57**	82.71	85.58
	Lavandula angustifolia		**9.59**	**23.30**	5.05
	Origanum vulgare		99.67	**9.33**	1.58
	Rosmarinus officinalis		**0.71**	**3.63**	3.17
	Salvia officinalis		**0.82**	**10.45**	8.11
	Thymus vulgaris		97.76	**7.61**	1.29
	Zingiber officinale		**0.19**	**23.00**	63.82

Essential oil combinations		Individual brine-shrimp mortality (%)*		Combined brine-shrimp mortality (%)*	ΣFIC**
**Essential oil 1**	**Essential oil 2**	**Essential oil 1**	**Essential oil 2**		
Lavandula angustifolia (lavender)	*Citrus aurantifolia*	**9.59**	**0.36**	**33.87**	48.51
	Citrus limon		**1.75**	**1.83**	*0.62*
	Citrus sinensis		**0.96**	**3.94**	2.27
	Commiphora molmol		**1.03**	81.68	44.00
	Melaleuca alternifolia		**1.07**	**4.31**	2.23
	Melaleuca viridiflora		**4.14**	70.66	12.21
	Origanum vulgare		99.67	**16.43**	*0.94*
	Pimenta racemosa		100.00	**5.90**	***0.34***
	Pinus sylvestris		**1.04**	**33.47**	17.86
	Rosmarinus officinalis		**0.71**	**9.18**	6.98
	Thymus vulgaris		97.76	76.90	4.40
Lavandula burnati (lavandin)	*Citrus limon*	**0.16**	**1.75**	**16.51**	57.72
	Citrus sinensis		**0.96**	**4.31**	16.09
Lavandula spica (lavender spike)	*Citrus limon*	**1.01**	**1.75**	**23.46**	18.33
	Citrus sinensis		**0.96**	**9.08**	9.26
Melaleuca cajuputii (cajeput)	*Citrus bergamia*	**0.60**	**1.22**	91.44	114.15
	Citrus limon		**1.75**	93.11	104.62
	Elettaria cardamonum		**0.00**	85.40	4341.77
	Eucalyptus globulus		**4.21**	90.75	86.84
	Helichrysum italicum		**9.50**	95.79	85.32
	Hyssopus officinalis		**6.65**	72.00	65.76
	Lavandula angustifolia		**9.59**	86.64	77.13
	Melaleuca viridiflora		**4.14**	80.45	77.14
	Myrtus communis		**0.00**	65.54	3331.96
	Pinus sylvestris		**1.04**	76.83	101.38
	Rosa damascena		97.32	79.58	67.11
	Rosmarinus officinalis		**0.71**	73.56	113.70
	Santalum austrocaledonicum		99.22	83.29	70.22
	Syzygium caryophyllata		99.57	83.06	70.03
	Thymus vulgaris		97.76	74.27	62.63
	Zingiber officinale		**0.19**	89.62	309.00
Melaleuca viridiflora (niaouli)	*Citrus sinensis*	**4.14**	**0.96**	76.26	49.13
	Mentha piperita		**6.98**	**28.43**	5.47
	Pinus sylvestris		**1.04**	83.30	50.16
	Rosmarinus officinalis		**0.71**	92.88	76.93
	Salvia officinalis		**0.82**	66.79	48.93
Mentha piperita (peppermint)	*Rosmarinus officinalis*	**6.98**	**0.71**	**31.37**	24.45
	Santalum austrocaledonicum		99.22	55.42	4.25
	Thymus vulgaris		97.76	77.57	5.96

Essential oil combinations		Individual brine-shrimp mortality (%)*		Combined brine-shrimp mortality (%)*	ΣFIC**
**Essential oil 1**	**Essential oil 2**	**Essential oil 1**	**Essential oil 2**		
Myrtus communis (myrtle)	*Melaleuca alternifolia*	**0.00**	**1.07**	**28.33**	1429.46
	Mentha piperita		**6.98**	59.98	3003.31
	Rosmarinus officinalis		**0.71**	84.74	4296.85
	Thymus vulgaris		97.76	78.91	3945.90
Ocimum basilicum (basil)	*Citrus aurantifolia*	**13.04**	**0.36**	**21.29**	30.19
	Citrus bergamia		**1.22**	76.68	34.40
	Cymbopogon citratus		100.00	**49.36**	2.14
	Eucalyptus globulus		**4.21**	**20.75**	3.26
	Hyssopus officinalis		**6.65**	**19.61**	2.23
	Juniperus virginiana		**0.57**	78.30	71.13
	Lavandula angustifolia		**9.59**	**9.33**	*0.84*
	Melaleuca alternifolia		**1.07**	**39.69**	19.99
	Melaleuca viridiflora		**4.14**	**20.05**	3.18
	Origanum vulgare		99.67	**18.69**	*0.81*
	Origanum vulgare		99.67	76.71	3.33
	Piper nigrum		**1.32**	73.80	30.79
	Rosmarinus officinalis		**0.71**	**25.06**	18.69
	Salvia officinalis		**0.82**	64.04	41.63
	Santalum austrocaledonicum		99.22	70.14	3.04
Origanum marjorana (marjoram)	*Citrus sinensis*	**0.34**	**0.96**	**4.74**	9.51
	Melaleuca alternifolia		**1.07**	**6.03**	11.76
	Rosmarinus officinalis		**0.71**	**2.15**	4.72
	Santalum austrocaledonicum		99.22	57.37	85.49
Origanum vulgare (origanum)	*Melaleuca alternifolia*	99.67	**1.07**	**7.75**	3.65
	Pinus sylvestris		**1.04**	**9.14**	4.44
	Rosmarinus officinalis		**0.71**	**3.59**	2.56
Pinus sylvestris (pine)	*Melaleuca alternifolia*	**1.04**	**1.07**	**26.91**	25.48
	Pimenta racemosa		100.00	87.51	42.57
	Rosmarinus officinalis		**0.71**	**42.28**	50.27
	Santalum austrocaledonicum		99.22	**49.86**	24.26
	Thymus vulgaris		97.76	79.10	38.48
Piper nigrum (black pepper)	*Citrus aurantifolia*		**0.36**	85.67	150.68
	Citrus bergamia		**1.22**	92.87	73.28
	Citrus limon		**1.75**	60.73	40.35
	Citrus sinensis		**0.96**	86.01	77.62
	Cupressus sempervirens		**1.02**	91.71	79.79
	Juniperus virginiana		**0.57**	90.35	112.85
	Lavandula angustifolia		**9.59**	88.34	38.07
	Origanum vulgare		99.67	74.70	28.67

Essential oil combinations		Individual brine-shrimp mortality (%)*		Combined brine-shrimp mortality (%)*	ΣFIC**
**Essential oil 1**	**Essential oil 2**	**Essential oil 1**	**Essential oil 2**		
	Rosmarinus officinalis		**0.71**	71.51	77.69
	Santalum austrocaledonicum		99.22	85.25	32.73
	Styrax benzoin		**2.47**	**1.18**	*0.69*
	Thymus vulgaris		97.76	94.59	36.32
Rosa damascena (rose)	*Santalum austrocaledonicum*	97.32	99.22	75.38	*0.77*
Rosmarinus officinalis (rosemary)	*Melaleuca alternifolia*	**0.71**	**1.07**	71.03	83.30
Salvia officinalis (sage)	*Melaleuca alternifolia*	**0.82**	**1.07**	53.28	57.38
	Santalum austrocaledonicum		99.22	78.42	48.38
	Thymus vulgaris		97.76	84.26	51.98
Salvia sclarea (clary sage)	*Boswellia carterii*	**0.20**	**1.39**	**18.34**	51.42
	Citrus aurantifolia		**0.36**	**20.93**	80.06
	Citrus bergamia		**1.22**	**44.88**	128.15
	Juniperus virginiana		**0.57**	56.47	187.20
	Lavandula angustifolia		**9.59**	**15.76**	39.36
	Lavandula burnati		**0.16**	**49.47**	279.78
	Rosa damascena		97.32	**9.63**	23.59
	Tagetes minuta		**1.30**	**12.64**	35.77
Santalum austrocaledonicum (sandalwood)	*Melaleuca alternifolia*	99.22	**1.07**	72.83	34.25
Styrax benzoin (benzoin)	*Boswellia carterii*	**2.47**	**1.39**	**3.87**	2.18
	Citrus bergamia		**1.22**	98.19	60.15
	Citrus limon		**1.75**	**24.97**	12.18
	Citrus sinensis		**0.96**	**19.61**	14.23
	Commiphora molmol		**1.03**	**16.41**	11.30
	Coriandrum sativum		**11.95**	80.90	19.75
	Cupressus sempervirens		**1.02**	**6.48**	4.50
	Lavandula angustifolia		**9.59**	**24.32**	6.19
	Mentha piperita		**6.98**	**44.80**	12.28
	Rosa damascena		97.32	**14.07**	2.92
	Santalum austrocaledonicum		99.22	**5.45**	1.13
Syzygium caryophyllata (clove)	*Cinnamonum zeylanicum*	99.57	98.49	**3.31**	***0.03***
	Citrus bergamia		**1.22**	**3.11**	1.29
	Citrus limon		**1.75**	**3.66**	1.06
	Citrus sinensis		**0.96**	**6.58**	3.48
	Commiphora molmol		**1.03**	**5.88**	2.89
	Cymbopogon citratus		100.00	**5.03**	***0.05***
	Eucalyptus globulus		**4.21**	**3.52**	***0.44***
	Juniperus virginiana		**0.57**	**4.47**	3.91
	Lavandula angustifolia		**9.59**	**2.30**	***0.13***

Essential oil combinations		Individual brine-shrimp mortality (%)*		Combined brine-shrimp mortality (%)*	ΣFIC**
**Essential oil 1**	**Essential oil 2**	**Essential oil 1**	**Essential oil 2**		
	Melaleuca alternifolia		**1.07**	**3.29**	1.55
	Mentha piperita		**6.98**	**3.06**	***0.23***
	Ocimum basilicum		**13.04**	**4.99**	***0.22***
	Pinus sylvestris		**1.04**	**2.23**	1.08
	Piper nigrum		**1.32**	**3.67**	1.41
	Rosmarinus officinalis		**0.71**	**3.44**	2.45
	Styrax benzoin		**2.47**	**7.15**	1.48
	Thymus vulgaris		97.76	**2.93**	***0.03***
	Zingiber officinale		**0.19**	**3.54**	9.26
Thymus vulgaris (thyme)	*Melaleuca alternifolia*	97.76	**1.07**	79.14	37.22
Zingiber officinale (ginger)	*Citrus limon*	**0.19**	**1.75**	**25.16**	72.84
	Citrus sinensis		**0.96**	**16.20**	50.77
	Commiphora molmol		**1.03**	**26.31**	81.47
	Cymbopogon citratus		100.00	83.94	219.50
	Melaleuca alternifolia		**1.07**	**29.10**	89.50
	Mentha piperita		**6.98**	**40.00**	107.30
	Pimenta racemosa		100.00	89.41	233.80
	Rosmarinus officinalis		**0.71**	**26.18**	86.87
	Salvia officinalis		**0.82**	82.65	266.29
	Santalum austrocaledonicum		99.22	91.79	240.04
	Thymus vulgaris		97.76	**9.06**	23.69
Positive control (Potassium dichromate)			99.95		
Negative control (Sea water)			0.00		

Brine-shrimp viability (%)* in bold denotes non-toxic effect (% mortality less than 50%); **ΣFIC**** in bold denotes synergistic effect (ΣFIC less than or equal to 0.50) and ΣFIC in italics denotes additive effect (ΣFIC greater than 0.50 and less than or equal to 1.00).
